# Unraveling the Photodynamic Activity of Cationic Benzoporphyrin-Based Photosensitizers against Bladder Cancer Cells

**DOI:** 10.3390/molecules26175312

**Published:** 2021-09-01

**Authors:** Ana T. P. C. Gomes, M. Graça P. M. S. Neves, Rosa Fernandes, Carlos F. Ribeiro, José A. S. Cavaleiro, Nuno M. M. Moura

**Affiliations:** 1 LAQV-REQUIMTE, Department of Chemistry, University of Aveiro, 3810-193 Aveiro, Portugal; gneves@ua.pt; 2 Coimbra Institute for Clinical and Biomedical Research (iCBR), Faculty of Medicine, University of Coimbra, 3000-548 Coimbra, Portugal; rcfernandes@fmed.uc.pt (R.F.); cribeiro@fmed.uc.pt (C.F.R.); 3 Center for Innovative Biomedicine and Biotechnology (CIBB), University of Coimbra, 3004-504 Coimbra, Portugal; 4 Center for Interdisciplinary Research in Health (CIIS), Faculty of Dental Medicine, Universidade Católica Portuguesa, 3504-505 Viseu, Portugal

**Keywords:** benzoporphyrin, photosensitizer, platinum complexes, cancer, photodynamic therapy

## Abstract

In this study, we report the preparation of new mono-charged benzoporphyrin complexes by reaction of the appropriate neutral benzoporphyrin with (2,2′-bipyridine)dichloroplatinum(II) and of the analogs’ derivatives synthesized through alkylation of the neutral scaffold with iodomethane. All derivatives were incorporated into polyvinylpyrrolidone (PVP) micelles. The ability of the resultant formulations to generate reactive oxygen species was evaluated, mainly the singlet oxygen formation. Then, the capability of the PVP formulations to act as photosensitizers against bladder cancer cells was assessed. Some of the studied formulations were the most active photosensitizers causing a decrease in HT-1376 cells’ viability. This creates an avenue to further studies related to bladder cancer cells.

## 1. Introduction

Cancer is a broad term that defines a group of diseases that can develop almost anywhere in the body, induced by the uncontrolled overgrowth of abnormal cells resulting in DNA mutations and consequently to the destruction of normal tissues. Malignant disorders are the second leading cause of death worldwide [1,2].

Currently, the most used treatment approaches are based on surgical resection of the tumoral mass, radio-, immuno-, and chemotherapy. However, these treatments display several disadvantages, such as severe radiation damage, limited applicability, lack of specificity, and severe side effects [2].

Concerning the chemotherapeutic approach, platinum-based drugs are the most used drugs against solid tumors such as bladder, testicular, ovarian, lung, neck, or head [3,4,5,6]. The mechanism of action of platinum-based drugs is based on their capability to bind DNA strands, which avoids the DNA strand from unzipping, by blocking the replication process, having as a consequence the malignant cell death [7,8,9,10,11].

In 1978, cisplatin was the first FDA-approved platinum-based drug to be used as an anticancer agent. After this milestone, other platinum-based drugs, namely carboplatin and oxaliplatin, were developed and also approved throughout the world [3,12]. Some others have regulatory approval only in some countries (e.g., nedaplatin, miriplatin, loboplatin, or heptaplatin) or are currently under clinical trials [9,13]. Despite the wide use of platinum-based drugs, the treated patients experienced severe side effects related to their poor selectivity for cancer cells. Among them are hematological and gastrointestinal toxicity, neuro-, nephro-, hepato-, or cardiotoxicity. However, intrinsic or acquired resistance cancer cells to platinum-based drugs is the major drawback related to the use of this class of compounds [3,7,8,11].

The scientific and medical communities are developing efforts to find efficient alternatives to chemotherapy as well as platinum-based drugs. Photodynamic therapy (PDT) is pointed out as one of the most promising approaches to be used for the treatment of malignant diseases [14,15]. PDT is a two-stage treatment that relies on the combined action of a photosensitizer (PS), molecular oxygen, and light. The PS is excited by light at a specific wavelength to an excited singlet state and by intersystem crossing to an excited triplet state that in the presence of dioxygen generates reactive oxygen species (ROS) by two mechanisms. The type I mechanism is promoted by electron transfer leading to radicals or radical ions species, while the type II mechanism involves energy transfer from PS to molecular oxygen producing singlet oxygen (^1^O_2_), pointed out as the prevalent process [16,17,18,19]. PDT displays various advantages when compared with chemotherapy; the PS is a non-toxic drug in the absence of light, is a non-invasive therapeutic procedure, and displays high selectivity for cancer cells and reduced long-term morbidity and resistance [16,20,21,22,23]. Moreover, this approach can be extended to non-oncological diseases, as well as non-clinical targets, including the photoinactivation of microorganisms [24,25,26,27,28,29].

Porphyrinoids are a class of compounds with distinctive structural, photochemical, and photophysical properties [30] to be used for a wide range of applications such as (chemo)sensors [31,32,33,34,35], (photo)catalysts [36,37,38,39,40,41], environmental protection [42,43,44], dyes for solar cells [45,46,47,48], and as PSs in medicine [21,49,50,51,52,53]. Regarding the latter use, porphyrinoids exhibit suitable features such as chemical and structural stability, good ^1^O_2_ generation capabilities, high fluorescence quantum yields, absorbance in the visible region of the electronic spectrum, no dark toxicity, and high affinity for cancer cells making them the most exploited class of compounds as PSs for PDT [15,54].

Concerning the modification of porphyrins and related compounds with platinum(II), most of the modifications reported in the literature describe the metalation of the tetrapyrrolic core with Pt(II) leading to the corresponding metalloporphyrinoid [55,56,57,58,59,60,61,62,63,64,65,66]. A literature survey also shows a considerable number of papers describing the preparation of porphyrin-platinum complexes by modification of peripheral moieties at *meso* positions, mainly by attachment of cyclometalated platinum units to pyridyl moieties [20,59,67,68,69,70,71,72,73]. Analogue synthetic approaches were also reported to modify corroles [74,75] and phthalocyanines [76,77,78,79,80]. 

Relatively less attention was paid to the preparation of porphyrin-platinum complexes throughout β-pyrrolic positions. Most of these studies were reported by Osuka’s group and involved the introduction of pyridyl units via metal-catalyzed cross-coupling reactions and further metalation with Pt(II) or Pt(IV) [81,82,83,84,85]. However, to the best of our knowledge, the preparation of benzoporphyrin-platinum complexes at the β-pyrrolic positions, namely at the isoindole-type unit, remains unexplored. 

Here, we describe the preparation of new mono-cationic benzoporphyrin complexes by reaction of the appropriate pyridyl benzoporphyrin scaffold with (2,2′-bipyridine)dichloroplatinum(II). With this strategy, it was intended to conjugate the antitumor activity of platinum derivatives with the well-known phototoxicity properties of porphyrin macrocycles. Moreover, positively charged analogues were also prepared through the *N*-alkylation of the benzoporphyrins pyridyl units with iodomethane to compare their properties/activity relationship. All the mono-charged synthesized benzoporphyrin derivatives were incorporated into polyvinylpyrrolidone (PVP) micelles, and the photosensitizer capability of the obtained formulations was evaluated and compared against a human bladder cancer cell line derived from transitional cell carcinoma. The PDT treatment of this type of cancer can benefit from the easy light delivery via insertion of a light source into urethra [86].

## 2. Results and Discussion

### 2.1. Synthesis

The synthesis of the positively monocharged benzoporphyrin derivatives **2** and **3** required the previous preparation of the scaffolds **1a,b** following procedures already reported by us. Briefly, 2-formyl-5,10,15,20-tetraphenylporphyrin reacts with the adequate 3- or 4-acetylpyridine in the presence of NH_4_OAc and catalytic amounts of La(OTf)_3_ in refluxing toluene for 4 h, under N_2_ atmosphere [87,88].

Then, the precursors **1a,b** reacted with (2,2′-bipyridine)dichloroplatinum(II) in refluxing CHCl_3_/MeOH (2:1) mixture for 24 h. After purification by column chromatography, the expected **2a** or **2b** derivatives were obtained in 68 and 87% yield, respectively. In the synthesis of the benzoporphyrin-platinum complex **2a**, it was recovered 18% of the starting benzoporphyrin **1a**. The lower reactivity of derivative **1a** and, consequently, the low yield obtained for **2a** are probably related with a hindrance effect due to the bulkiness of the (2,2′-bipyridine)chloroplatinum(II) moiety and the proximity of the nitrogen atom of the pyridyl unit to the benzoporphyrin core. An extension of the reaction time from 24 to 48 h led to a slight improvement in the yield of benzoporphyrin-platinum complex **2a** to 72%. 

It is worth noting that all the attempts to prepare the analog benzoporphyrin-platinum complex bearing a 2-substituted pyridyl moiety failed, probably due to an even higher steric hindrance effect induced by the bulkiness of the (2,2′-bipyridine)chloroplatinum(II) moiety and the proximity of the pyridyl nitrogen atom with the benzoporphyrin core.

**Scheme 1:**
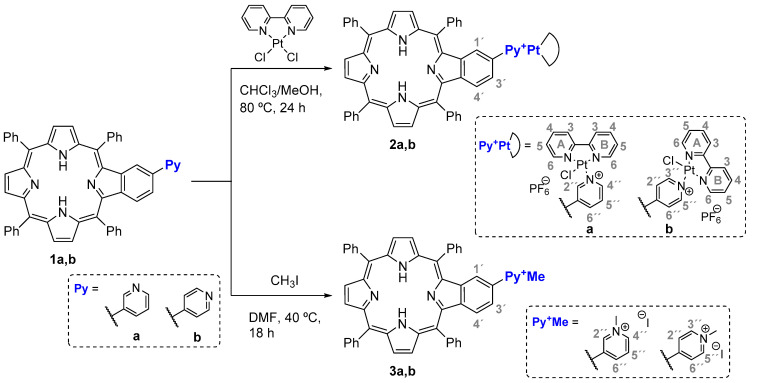
Synthetic approach to prepare benzoporphyrins **2a,b** and **3a,b**.

Compounds **3a** and **3b** were prepared in 97 and 98% yield, respectively, from the corresponding neutral derivative **1a**,**b** by alkylation reaction with iodomethane in DMF at 40 °C for 24 h (Scheme 1). This is a typical and well-established protocol to prepare porphyrinoids bearing pyridinium moieties and, once again, it revealed to be effective for preparing the benzoporphyrin derivatives **3a,b**.

The structures of compounds **2a,b** and **3a,b** were confirmed by NMR spectroscopy and mass spectrometry data (see Figures S1–S22. The mass spectra of the mono-charged benzoporphyrin derivatives **2a,b** and **3a,b** exhibit the *m/z* peak corresponding to the [M + 2H]^+^ or [M + 2H]^+●^ molecular ion. However, it is important to point out that, for all the compounds synthesized, the corresponding [M + 2H]^+^ and [M + 2H]^+●^ species are formed in the gas phase due to the reduction in one of the β-pyrrolic positions. Similar results were already observed by us in a previous publication [89].

The ^1^H NMR analysis of the derivatives **2a,b** and **3a,b** supports also the proposed structures with the resonances of six β-pyrrolic protons appearing in the aromatic region, between δ 8.98 and δ 8.60 ppm. In all the ^1^H NMR spectra, the distinguishing singlet at ca. δ −2.7 ppm generated by the resonance of the *N*-H protons from the free-base benzoporphyrin core is also observed.

The ^1^H NMR spectra of compound **3a,b** show a singlet at around δ 4.67 ppm generated by the resonances of the methyl groups’ protons confirming the formation of the pyridinium moiety. All the remaining signals generated by the resonances of the protons from the benzoporphyrin moieties, as well as from the phenyl ring at the *meso*-positions appear in the aromatic region, being the most deshielded signals generated by the protons near the nitrogen atom at the pyridinium unity. 

In the ^1^H NMR of compounds **2a,b**, the most deshielded signals (ca. 9.6 ppm) are due to the resonances of the protons from the 6 positions of the 2,2′-bipyridine unit, while the protons from the pyridyl units are shielded by the presence of the platinum core when compared with the ones from the neutral precursors. The signals generated by the remaining protons from the benzoporphyrin core are not significantly affected by the 2,2′-bipyridine)chloroplatinum(II) unit and display similar chemical shifts to the ones observed for the corresponding **3a,b** derivatives.

**Figure 1:**
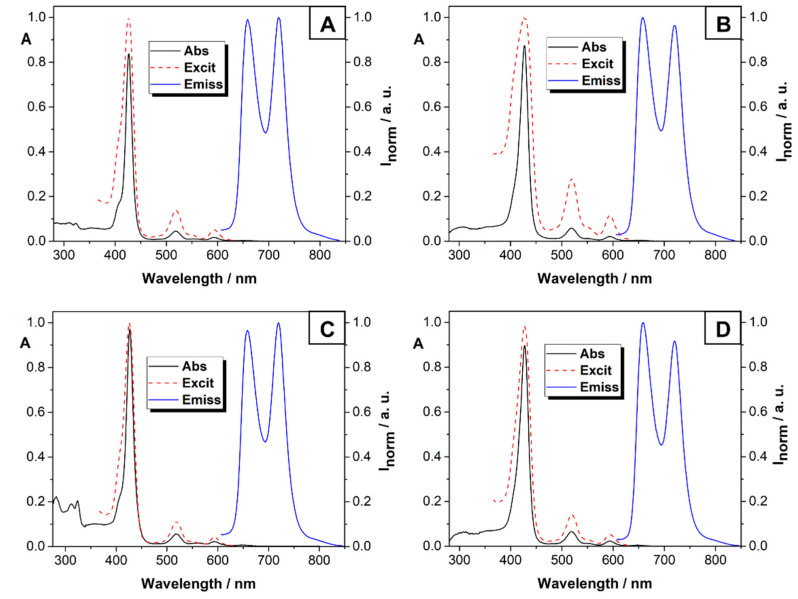
Absorption and normalized emission and excitation spectra of **2a** (**A**), **3a** (**B**), **PVP-2a** (**C**) and **PVP-3a** (**D**) in DMF at 298 K. ([**2a**] = [**3a**] = [**PVP-2a**] = [**PVP-3a**] = 3.0 × 10^−6^ M; λ_ex**2a**_ = λ_ex**3a**_ = 593 nm and λ_ex**PVP-2a**_ = λ_ex**PVP-3a**_ = 595 nm; λ_em**2a**_ = λ_em**3a**_ = 659 nm and λ_em**PVP-2a**_ = λ_em**PVP-3a**_ = 658 nm).

The absorption, steady-state fluorescence emission, and excitation spectra of the new derivatives were recorded in *N,N*-dimethylformamide (DMF) solution at 298 K. The UV-Vis spectra of compounds **2a,b** and **3a,b** were not significantly affected by the modification performed into the benzoporphyrinic ring, presenting the typical features of free-base porphyrin derivatives due to π–π* transitions [90,91,92]. Both series **2a,b** and **3a,b** exhibit a strong Soret band at ca. 427 nm assigned to allowed S_0_ → S_2_ transitions and two Q bands at approximately 520 and 595 nm due to S_0_ → S_1_ transitions. Additionally, absorption bands centered from 282 to 324 nm were observed for derivatives **2a,b** due to the ligand to metal charge transfer (LMCT) transition from the bipyridine moiety to the platinum ion. The fluorescence emission spectra of compounds **2** and **3** obtained after excitation at approximately 595 nm present two bands centered at ca. 660 and 720 nm (Table 1, Figure 1A,B and Figure S23A,B).

The resemblance between the absorption and excitation spectra rules out the presence of emissive impurities. The large Stokes shift (ca. 66 nm) displayed by both prepared series of benzoporphyrin derivatives are indicative of a change in the electronic nature of the excited state compared with that of the ground state. The fluorescence quantum yields (Φ_F_) determined by the internal reference method with respect to a solution of 5,10,15,20-tetraphenylporphyrin (TPP) in DMF as standard (Φ_F_ = 0.11) [93,94] are shown in Table 1. The Φ_F_ values range from 0.05 to 0.07, and no noticeable differences were induced by the presence of the cyclometalated (2,2′-bipyridine)platinum(II) moieties or the methyl groups.

### 2.2. Incorporation into PVP Micelles

Benzoporphyrin derivatives **2** and **3** were used to prepare polyvinylpyrrolidone (PVP) formulations aiming to avoid aggregation phenomena in aqueous medium due to their low hydrophilic character (miLog *P*: 8.45–9.91) [95]. This is a low-cost approach that requires the dissolution of both PS and *N*-vinylpyrrolidone (VPD) in CHCl_3_ solution, stirring the resulting mixture for 2 h at room temperature, and then solvent removal under a nitrogen flow. The resulting residue, after being maintained for 48 h at 40 °C, was dissolved in water and submitted to dialysis affording the expected PVP-PS formulations **PVP-2a,b** and **PVP-3a,b**. It is worth noting that the PVP-PS formulations prepared retained the photophysical features previously discussed for PS **2** and **3** without noticed changes due to their incorporation into PVP micelles (Table 1, Figure 1C,D and Figure S23C,D).

VPD was selected as the monomer to prepare PVP formulations due to their already reported features, namely, pharmacokinetic and pharmacological properties, non-toxicity, and water-solubility of the obtained micelles [96]. This strategy allows to improve the hydrophilicity of biologically active drugs and is being efficiently used to solubilize neutral porphyrin-base PS in water with positive effects in photodynamic processes [97,98,99,100]. Moreover, this carrier demonstrated to be non-toxic for both normal and cancer cells after PDT treatment [100]. However, to the best of our knowledge, this approach has not been used with benzoporphyrin-type derivatives.

### 2.3. Photostability and Singlet Oxygen Generation

Photostability is a relevant parameter to evaluate the PS potential to be used in photodynamic processes such as PDT. The photostability assays for PVP-PS formulations **PVP-2a,b** and **PVP-3a,b** were performed in PBS by monitoring the Soret band decay (λ_max_ = 425 nm) after irradiation with white light at an irradiance of 20 mW·cm^−2^ for different irradiation periods. After 30 min of irradiation, formulations **PVP-2b** and **PVP-3a,b** showed a Soret band absorption decay ranging from 11 to 16%, while, for **PVP-2a**, the decrease was 28% (Table S1). As such, it is possible to conclude that the two synthetic strategies used to modify the benzoporphyrin core and the incorporation of the obtained benzoporphyrin derivatives into PVP micelles allowed to afford PVP formulations with adequate photostability.

**Figure 2:**
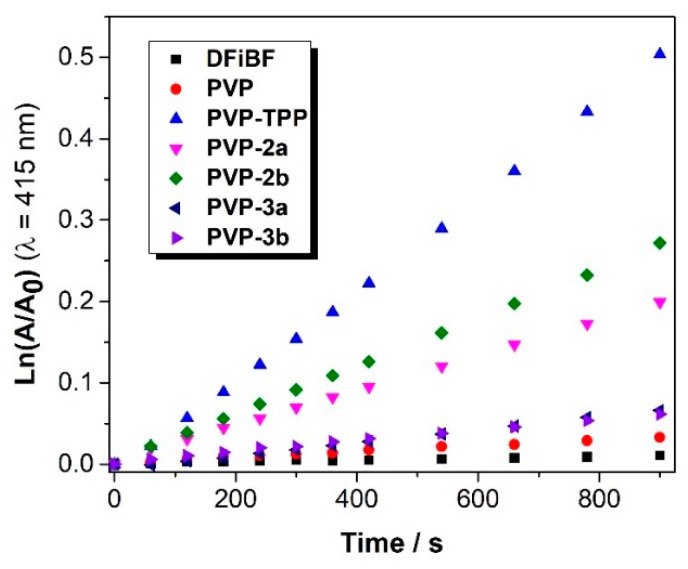
Time-dependent photodecomposition of DPiBF at 50 µM photosensitized by PVP-PS formulations **PVP-2a,b**, **PVP-3a,b**, and **PVP-TPP** at 0.5 µM upon irradiation with red light (630 ± 20 nm) at an irradiation of 11 mW·cm^−2^.

Besides photostability, another relevant feature for a PS to be used in PDT is its capability to generate ROS, namely, singlet oxygen (^1^O_2_) [101]. The generation of ^1^O_2_ by the PVP-PS formulations was qualitatively determined by monitoring at 415 nm, the photooxidation of the ^1^O_2_ quencher 1,3-diphenylisobenzofuran (DPiBF) to the colorless *o*-dibenzoylbenzene, after the Diels–Alder-like reaction [102,103,104]. The irradiations of each PVP-PS formulation in DMF and in the presence of dioxygen were performed at a fluence of 11 mW·cm^−2^ and the results obtained from the DPiBF time-dependent photodecomposition are summarized in Figure 2.

It is worth noting that, for these assays, a **PVP-TPP** formulation was prepared to be used as reference, since 5,10,15,20-tetraphenylporphyrin (**TPP**) is pointed out as a good singlet oxygen generator [105].

The **PVP-2a,b** formulations showed to be better ^1^O_2_ generators than **PVP-3a,b** formulations, despite both series displaying worse capability than the one presented by the **PVP-TPP** formulation. The capability of **PVP-3a,b** formulations is 10% of the one exhibited by the reference and is not significantly affected by the position of the charge in the pyridinium unit. The ability of the **PVP-2a** and **PVP-2b** formulations to produce ^1^O_2_ is 60 and 45% lower when compared with the reference, respectively, but even so, it is 4- to 5-fold higher than the ones presented by the **PVP-3a,b** formulations. Yet, for the **PVP-2a,b** formulations, the position of the charge and the (2,2′-bipyridine)chloroplatinum(II) unit influences the PS production of ^1^O_2_, with **2b** being the one with the better yield of ^1^O_2_.

From the analysis of Figure 2, it is obvious that the absorbance of DPiBF, when irradiated in the absence of a PS, remains almost unchanged, as well as in the presence of just PVP. These results revealed the potential of the PVP-PS formulations prepared to be used in PDT and prompted us to evaluate their efficiency as PSs against bladder cancer cells.

## 3. Photodynamic Activity of PVP-2a,b and PVP-3a,b Formulations against Human Bladder Cancer Cells

### 3.1. Cellular Uptake of ***PVP-2a,b*** and ***PVP-3a,b*** Formulations

**Figure 3:**
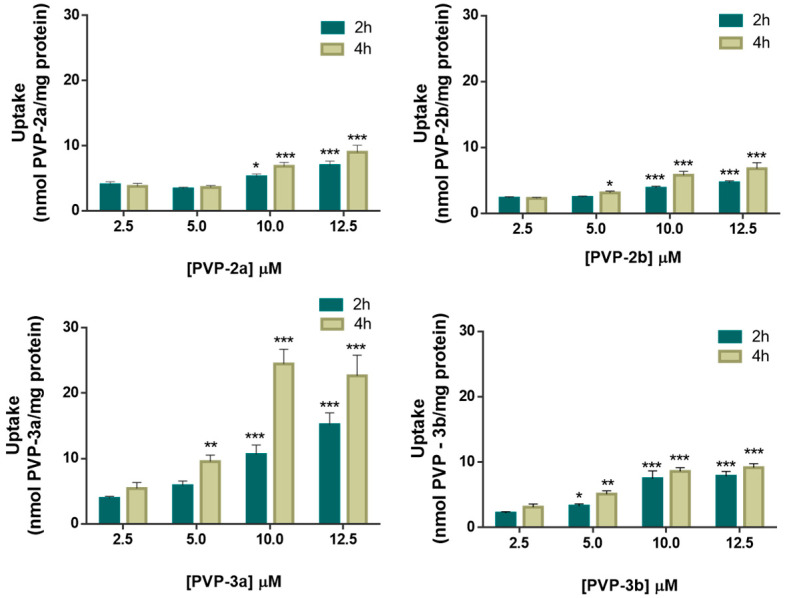
Intracellular uptake of formulations **PVP-2a,b** and **PVP-3a,b** by HT-1376 cells. Data are the mean ± S.D. of at least three independent experiments performed in triplicates: * (*p* < 0.05), ** (*p* < 0.01), *** (*p* < 0.001) significantly different from uptake of PVP formulations at lower concentration.

The ability of PSs **PVP-2a,b** and **PVP-3a,b** for being internalized by cancer cells was spectrofluorometrically evaluated, using a human bladder cancer cell line derived from transitional cell carcinoma (HT-1376 cell line). Thus, HT-1376 cells were incubated in the dark with increasing concentrations of each PVP formulation (2.5, 5.0, 10.0, and 12.5 μM) in PBS for 2 and 4 h. The results for the intracellular uptake of **PVP-2a,b** and **PVP-3a,b** are presented in Figure 3 and show that the internalization of the PVP formulations in HT-1376 cell line is, in general, concentration and time-dependent, reaching the maximum after 4 h of incubation. It is also evident that **PVP-3a** formulation presents the highest intracellular accumulation, 24.5 ± 2.24 and 22.6 ± 3.17 nmol of PS/mg of protein, for 10 and 12.5 µM, respectively, and after 4h. Although absolute values are lower, the maximum intracellular accumulation also occurs after 4 h incubation for formulations **PVP-3b** and **PVP-2a,b.**

**PVP-2b** presents the lowest internalized PS concentration, with 5.77 ± 0.59 and 6.78 ± 0.89 nmol of PS/mg of protein, for 10 and 12.5 μM, respectively. The intracellular accumulation in HT-1376 cells of **PVP-2a** and **PVP-3b** reach the maximum at 10 μM after 4 h of incubation, reaching a value of 9.02 ± 1.08 and 9.16 ± 0.61 nmol of PS/mg of protein. 

### 3.2. Cell Viability after PDT Treatment with ***PVP-2a,b*** and ***PVP-3a,b*** Formulations

**Figure 4:**
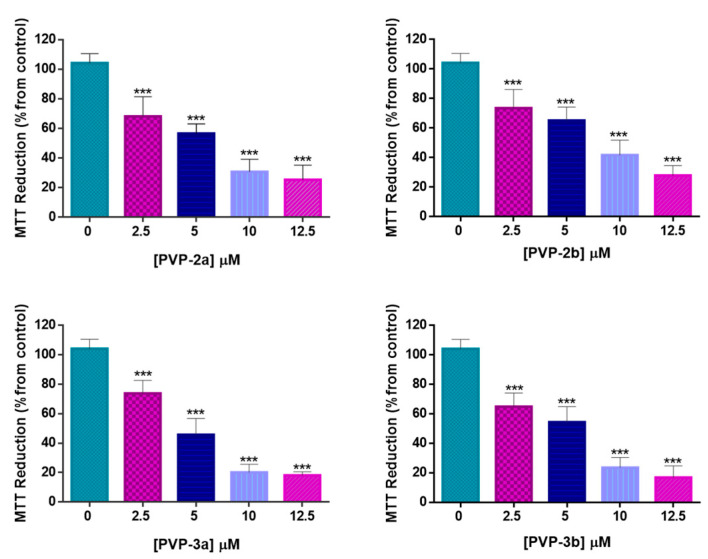
Phototoxic effect of formulations **PVP-2a,b** and **PVP-3a,b** in HT-1376 cells. Cells were incubated with the formulations for 4 h and then irradiated with white light (fluence rate of 20 mW·cm^−2^) for 40 min. Cytotoxicity was evaluated 24 h after PDT. Data are the mean value ± S.D. of at least three independent experiments performed in triplicates: * (*p* < 0.05), ** (*p* < 0.01), *** (*p* < 0.001) compared to MTT reduction (%) at 24 h after PDT for control cells (untreated cells).

The photodynamic effect of the **PVP-2a,b** and **PVP-3a,b** formulations was evaluated in bladder cancer cell line HT-1376 at 2.5, 5.0, 10.0, and 12.5 μM. The cell line was incubated in the dark for 4 h with the PVP formulations and then irradiated with white light for 40 min with an irradiance of 20 mW·cm^−2^. The cell viability was accessed by the MTT colorimetric assay after 24 h of PDT protocol. The results obtained are presented in Figure 4, and the IC50_PDT_ values (for a fluence rate of 20 mW·cm^−2^) of all formulations are plotted in Table 2.

The results showed that all PVP formulations caused a decrease in the HT-1376 cell viability, being possible to observe that the phototoxicity increased with the PS concentration**. PVP-3a,b** formulations showed to be the most active PSs causing a decrease in HT-1376 cell viability higher than 80% for the maximum concentration. This superior efficiency is also proved by their lower IC50_PDT_ values (5.58 and 5.51 μM for **PVP-3a** and **PVP-3b**, respectively). Although also highly efficient, the phototoxicity values of **PVP-2a,b** formulations were lower, causing a reduction in the HT-1376 cell viability of around 70% for the highest concentration. This fact could be explained by the lower **PVP-2a,b** accumulation inside HT-1376, when compared with the internalization of **PVP-3a,b** into the bladder cancer cell line.

The same protocol without the irradiation procedure was performed to evaluate the cytotoxic effect of all formulations. As expected, no cytotoxicity was observed in cells incubated with **PVP-2a,b** and **PVP-3a,b** in the dark for at least 24 h (data shown in Figure S24). 

It is well known that the efficiency of PDT depends on the intrinsic efficacy of the PS, and there are many PS properties that need to be taken into account, such as ^1^O_2_ generation, aggregation, and photodegradation behavior [106]. Although being the least efficient in the ^1^O_2_ generation, it is noteworthy that formulations **PVP-3a,b** were the ones that demonstrated higher photostability and higher internalization into the cancer cell line. The conjugation of these important properties could explain the higher efficiency of these formulations in the decrease in the HT-1376 cell viability after the PDT procedure. 

Moreover, it is also important to note that, in this particular case, the insertion of a 2,2’-bipyridine-platinum moiety into the benzoporphyrin macrocycle did not enhance the PDT effect in the cancer cell line.

## 4. Materials and Methods

### 4.1. General Remarks

^1^H and ^13^C NMR spectra were recorded on a Bruker Avance 300 spectrometer at 300.13 MHz and a Bruker Avance 500 spectrometer at 500.12 and 125.77 MHz, respectively. CDCl_3_ was used as solvent and tetramethylsilane (TMS) as internal reference. Chemical shifts are expressed in *δ* (ppm) and the coupling constants (*J*) are expressed in Hertz. HRMS were recorded on a VG AutoSpec M mass spectrometer using MeOH as solvent and 3-nitrobenzyl alcohol (NBA) as matrix. The UV–Vis spectra were recorded on an UV-2501 PC Shimadzu spectrophotometer using DMF as solvent. Fluorescence emission spectra were recorded on a Horiba Jobin-Yvon Fluoromax 3 spectrofluorometer and fluorescence quantum yields of compounds **2a,b** and **3a,b** and PVP-PS formulations **PVP-2a,b** and **PVP-3a,b** were measured by using a solution of TPP in DMF as a standard (Φ_F_ = 0.11). Flash chromatography was carried out using silica gel (230–400 mesh), and preparative thin-layer chromatography was carried out on 20 × 20 cm glass plates coated with silica gel (1 mm thick). The reactions were routinely monitored by thin-layer chromatography (TLC) on silica gel precoated F254 Merck plates.

### 4.2. Synthesis

#### 4.2.1. Synthesis of the Benzoporphyrin Precursors 1

Precursors **1a** and **1b** were synthesized according to the previous procedures described. The structures of both porphyrin-based PS were confirmed by ^1^H-NMR spectroscopy and mass spectrometry, and the data are in accordance with the data reported [87].

#### 4.2.2. Synthesis of Porphyrin-Platinum(II) Complexes

(2,2′-Bipyridine)dichloroplatinum(II) (13.7 mg, 32.4 µmol) was added to a solution of the adequate benzoporphyrin **1a** or **1b** (20 mg, 27 µmol) in a CHCl_3_/MeOH mixture (2:1, 1.5 mL) in a sealed tube. The reaction mixture was stirred at 100 °C for 24 h. Then, 0.2 M aqueous saturated solution of KPF_6_ was added to the reaction mixture, and the precipitate obtained, corresponding to the PF_6_^−^ salt, was filtered, dissolved in CH_2_Cl_2_, and washed with distilled water, and the organic layer was collected. The solvent was evaporated under reduced pressure, and the crude purified by column chromatography using CH_2_Cl_2_/MeOH (98:2) as the eluent. The benzoporphyrin-platinum(II) complexes **2a** and **2b** were obtained pure after crystallization from CH_2_Cl_2_/hexane.

Compound **2a**: Yield: 68%. ^1^H NMR (500 MHz, CDCl_3_): *δ* 9.66 (1H, dd, *J* = 1.0 and 5.6 Hz, H-6A), 9.60 (1H, dd, *J* = 1.2 and 5.7 Hz, H-6B), 9.09 (1H, d, *J* = 2.0 Hz, H-2″), 8.94 (1H, d, *J* = 5.0 Hz, H-β), 8.89 (1H, d, *J* = 5.0 Hz, H-β), 8.87 (1H, d, *J* = 5.0, H-β), 8.81–8.79 (1H, m, H-4″), 8.75–8.70 (3H, m, H-β), 8.43–8.39 (2H, m, H-6″ and H-*o*-Ph), 8.36 (1H, d, *J* = 8.0 Hz, H-*o*-Ph), 8.32–8.28 (2H, m, H-3B and H-*o*-Ph), 8.24–8.16 (6H, m, H-3a, H-4B and H-*o*-Ph), 8.09 (1H, d, *J* = 7.7 Hz, H-*o*-Ph), 8.02 (1H, td, *J* = 1.6 and 7.8 Hz, H-4A), 7.94–7.73 (12H, m, H-1’, H-3’ and H-*m,p*-Ph), 7.70–7.66 (H-5″ and H-*m*-Ph), 7.57–7.54 (1H, m, H-5B), 7.48–7.45 (1H, m, H-5A), 7.40–7.36 (1H, m, H-*p*-Ph), 7.34 (1H, d, *J* = 8.1 Hz, H-4′), −2,73 (2H, s, *N*H) ppm. ^13^C NMR (125 MHz, CDCl_3_): *δ* 159.8, 157.0, 156.5, 156.0, 155.2, 152.2, 151.3, 150.6, 149.7, 147.9, 147.7, 143.0, 141.9, 141.7, 139.2, 138.6, 134.5, 134.4, 134.3, 134.0, 133.6, 133.5, 133.2, 132.3-129.8 (C-β), 129.1, 128.9, 128.4, 128.3, 128.04, 127.97, 127.9, 127.7, 127.2, 127.1, 127.0, 126.9, 126.8, 126.5, 126.4, 125.1, 124.3, 122.5, 121.5, 118.1, 117.5, 117.2 ppm. MS (ESI(+)) = 1129.3 [M]^+^. V-Vis (DMF): λ_max_ (log ε) 286 (4.44), 313 (4.43), 323 (4.45)427 (5.45), 518 (4.01), 593 (3.57) nm. MS-ESI(+): 1129.4 [M + 2H]^+^. HRMS-ESI(+): *m/z* calcd. for C_63_H_45_ClN_7_Pt 1129.2916 [M + 2H]^+^; found 1129.2963.

Compound **2b**: Yield: 87%. ^1^H NMR (500 MHz, CDCl_3_): δ 9.85 (1H, dd, *J* = 0.9 and 5.9 Hz, H-6B), 9.59 (1H, dd, *J* = 0.9 and 5.6 Hz, H-6A), 8.95 (1H, d, *J* = 5.0 Hz, H-β), 8.91 (1H, d, *J* = 5.0 Hz, H-β), 8.87 (1H, d, *J* = 5.0 Hz, H-β), 8.80 (1H, d, *J* = 4.9 Hz, H-β), 8.76 (2H, d, *J* = 6.6 Hz, H-2″ and H-6″), 8.73–8.70 (2H, m, H-β), 8.42 (1H, d, *J* = 8.0 Hz, H-*o*-Ph), 8.37–8.34 (2H, m, H-*o*-Ph), 8.29–8.18 (8H, m, H-4A, H-3B, H-*o*-Ph), 8.16 (1H, td, *J* = 1.4 and 7.9 Hz, H-4B), 7.98–7.95 (4H, m, H-3′, H-3″, H-5″ and H-3A), 7.93–7.74 (11H, m, H-*m,p*-Ph and H-1′), 7.69–7.66 (1H, m, H-5B), 7.64–7.61 (2H, m, H-*p*-Ph and H-5A), 7.36 (1H, d, *J* = 8.4 Hz, H-4’), −2.70 (2H, s, *N*H) ppm. ^13^C NMR (125 MHz, CDCl_3_): δ 159.9, 156.8, 156.1, 155.2, 152.6, 150.8, 150.7, 150.1, 148.0, 147.6, 146.9, 146.0, 143.2, 141.8, 141.7, 141.6, 139.2, 138.3, 135.5, 134.5, 134.4, 134.3, 133.5, 133.0, 132.3–129.63 (C-β), 129.3, 129.14, 129.12, 129.0, 128.5, 128.3, 128.1, 128.0, 127.9, 127.3, 127.2, 126.9, 126.84, 126.79, 124.9, 124.8, 124.1, 123.0, 122.4, 121.6, 121.5, 118.2, 117.9, 117.6 ppm. UV-Vis (DMF): λ_max_ (log ε) 312 (4.67), 324 (4.43), 427 (5.49), 520 (4.32), 594 (3.87) nm. MS-ESI(+): 1129.3 [M + 2H]^+^. HRMS-ESI(+): *m/z* calcd. for C_63_H_45_ClN_7_Pt 1129.2916 [M + 2H]^+^; found 1129.2926.

#### 4.2.3. Synthesis of Cationic Benzoporphyrins **3a**,**b**

The appropriate neutral benzoporphyrin **1a,b** (20 mg, 27 µmol) was dissolved in DMF (1.0 mL) and to the solution was added an excess of iodomethane (0.1 µL, 1.6 mmol). The resulting mixture was stirred at 40 °C for 18 h, and, after this period, diethyl ether was added. The precipitate obtained was filtered through a cotton pad and washed with diethyl ether. Then, the solid was dissolved with a CH_2_Cl_2_/MeOH (95:5) mixture, the solvent evaporated, and the expected compounds **3a** and **3b** were obtained in almost quantitative yield, after hexane/CH_2_Cl_2_ crystallization.

Compound **3a**: Yield: 98%. ^1^H NMR (300 MHz, CDCl_3_): *δ* 9.32 (1H, dd, *J* = 5.9 Hz, H-4″), 8.89-8.79 (4H, m, H-β), 8.66–8.60 (3H, H-β and H-2″), 8.17–8.11 (8H, m, H-*o*-Ph), 7.98–7.96 (1H, m, H-5″), 7.92 (2H, d, *J* = 8.4 Hz, H-3′), 7.88 (1H, d, *J* = 7.6 Hz, H-6″), 7.81–7.66 (13H, m, H-*m,p*-Ph and H-1′), 7.33 (1H, d, *J* = 8.4 Hz, H-4′), 4.66 (3H, s, -CH_3_) −2.76 (2H, s, *N*H) ppm. ^13^C NMR (125 MHz, CDCl_3_): *δ* 159.7, 155.4, 146.3, 145.8, 145.6, 144.5, 143.6, 142.8, 141.9, 141.7, 141.6, 140.0, 138.4, 138.3, 135.4, 134.53, 134.49, 134.4, 133.6, 133.5, 129.1, 128.3, 128.2, 128.1, 128.01, 127.98, 127.95, 127.2, 126.9, 121.72, 121.65, 118.3, 117.7, 117.2, 49.6 ppm. UV-Vis (DMF): λ_max_ (log ε) 427 (5.39), 518 (4.07), 593 (3.72) nm. MS-ESI(+): 757.5 [M + 2H]^+●^. HRMS-ESI(+): *m/z* calcd. for C_54_H_39_N_5_ 757.3122 [M + 2H]^+●^; found 757.3094.

Compound **3b**: Yield: 97%. ^1^H NMR (300 MHz, CDCl_3_): *δ* 8.98–8.92 (4H, m, H-β and H-3″,5″), 8.85 (1H, d, *J* = 5.0 Hz, H-β), 8.81 (1H, d, *J* = 5.0 Hz, H-β), 8.73 (2H, s, H-β), 8.39 (2H, d, *J* = 6.9 Hz, H-2″,6″), 8.24–8.19 (6H, m, H-*o*-Ph), 8.15 (2H, d, *J* = 6.8 Hz, H-*o*-Ph), 8.09 (1H, d, *J* = 8.5Hz, H-3′), 8.04–7.93 (3H, m, H-*m,p*-Ph), 7.88–7.72 (10H, m, H-*m,p*-Ph and H-1′), 7.37 (1H, d, *J* = 8.5 Hz, H-4′), 4.67 (3H, s, -CH_3_), −2.64 (2H, s, *N*H) ppm. ^13^C NMR (125 MHz, CDCl_3_) *δ* 159.9, 155.5, 155.4, 155.0, 147.1, 146.5, 145.7, 145.5, 144.8, 143.1, 141.64, 141.60, 141.57, 140.0, 138.8, 138.5, 136.1, 134.5, 134.3, 133.4, 133.2, 129.2, 128.6, 128.4, 128.2, 128.1, 128.0, 127.6, 127.3, 126.90, 126.87, 126.6, 125.2, 121.8, 121.6, 119.1, 118.5, 117.6, 49.1 ppm. UV-Vis (DMF): λ_max_ (log ε) 429 (5.06), 522 (4.15), 597(3.74) nm. MS ESI(+): 757.3 [M + 2H]^+●^. HRMS-ESI(+): *m/z* calcd. for C_54_H_39_N_5_ 757.3122 [M + 2H]^+●^; found 757.3129.

### 4.3. General Procedure to Prepare PVP-PS Micelles

Chloroform solutions of *N*-vinylpyrrolidone (100 mg) and compounds **2a,b** or **3a,b** (10% *w*/*w*) were mixed in a Becker and the solution stirred for 2 h at room temperature for a full homogenization. Then, the solvent was evaporated under nitrogen flow and the reddish-brown solid obtained was dried in an oven at 40 °C for 48 h. The resulting residues were dissolved in 2 mL of water and submitted to dialysis in distilled water at pH 7. After this approach, **PVP-2a,b** and **PVP-3a,b** formulations were obtained.

### 4.4. Photostability Assays

In a glass cuvette, we prepared PBS solutions of PVP-based formulations **PVP-2a,b** and **PVP-3a,b** (5.0 µM), which were kept in the dark at room temperature. Then, the solutions were irradiated with white light (400–750 nm) using a light emission diode (LED) system (ELMARK—VEGA20, 20 W, 1400 lm) with an irradiance of 25 mW·cm^−2^, for 30 min. The absorption spectra were recorded at 0, 1, 2, 3, 4, 5, 10, 20, and 30 min after irradiation. 

### 4.5. Singlet Oxygen Generation

To evaluate the ability of PVP formulations (**PVP-2a,b**, and **PVP-3a,b**) to generate singlet oxygen (^1^O_2_), in a 1 × 1 cm cuvette, we prepared 3 mL solutions, each one containing a PS (0.5 µM) and DPiBF (50 µM) in DMF. The solutions were irradiated with a red-light LED board (630 ± 20 nm) at an irradiance of 11 mW·cm^−2^ for 15 min at room temperature under gentle magnetic stirring. Control assays using a DPiBF solution at 50 µM and the **PVP** and **PVP-TPP** formulations (0.5 µM) and just a DPiBF (50 µM) were also performed.

### 4.6. Photodynamic Activity of ***PVP-2a,b*** and ***PVP-3a,b*** Formulations against Human Bladder Cancer Cells 

The study of the PDT efficiency of **PVP-2a,b** and **PVP-3a,b** formulations was conducted using a human bladder cancer cell line HT-1376 derived from high-grade transitional cell carcinoma (from the American Type Culture Collection, ATCC, Manassas, VA, USA). This cell line was cultured in Roswell Park Memorial Institute medium (RPMI-1640) supplemented with 10% (*v*/*v*) of fetal bovine serum (Life Technologies, Carlsbad, CA, USA), 100 U/mL penicillin, 100 mg/mL streptomycin and 0.25 mg/mL amphotericin B (Sigma, Darmstadt, Germany).

#### 4.6.1. Cellular Uptake of **PVP-2a,b** and **PVP-3a,b** Formulations

For the determination of the cellular uptake of **PVP-2a,b** and **PVP-3a,b** formulations, HT-1376 cells were seeded (9.4 × 10^4^ cells.cm^−2^) in 96-well cell culture plates and maintained in culture medium under an air atmosphere containing 5% of CO_2_. After seeding the cells overnight, they were washed twice with PBS and incubated for 2 h and 4 h in darkness (at 37 °C under air atmosphere containing 5% of CO_2_) with **PVP-2a,b** and **PVP-3a,b** at 2.5, 5.0, 10.0, and 12.5 μM concentrations. HT-1376 cells were immediately washed with PBS and lysed in 1% *m*/*v* sodium dodecyl sulfate (SDS; Sigma) in PBS. **PVP-2a,b** and **PVP-3a,b** intracellular concentration was determined by spectrofluorimetry using a microplate reader Synergy HT, BioTek, Winooski, VT, USA, equipped with excitation/emission wavelengths set at 360 nm/675 nm. The results were normalized for protein concentration (determined by bicinchoninic acid reagent; Pierce, Rockford, IL, USA).

#### 4.6.2. Cell Viability after PDT Treatment with **PVP-2a,b** and **PVP-3a,b** Formulations

HT-1376 cells were seeded (9.4 × 10^4^ cells.cm^−2^) in 96-well cell culture plates and maintained in culture medium under an air atmosphere containing 5% of CO_2_ overnight. The cells were washed twice with PBS and incubated with 2.5, 5.0, 10.0, and 12.5 μM of **PVP-2,ab** and **PVP-3a,b** formulations for 4 h in the dark. The cells were then washed twice with PBS and covered with 100 μL of fresh medium. The cells were irradiated for 40 min with white light delivered by an illumination system (LC-122 LumaCare, London, UK) equipped with a halogen/quartz 250 W lamp coupled to the selected interchangeable optic fiber probe (400–800 nm) at a fluence rate of 20 mW·cm^−2^. After irradiation, the cells were incubated in a humidified incubator with 5% of CO_2_ atmosphere and 95% of air. After 24 h of the PDT protocol, cell phototoxicity was determined by measuring the ability of cancer cells to reduce 3-[4,5-dimethylthiazol-2-yl]-2,5-diphenyl-tetrazolium bromide (MTT, Sigma), to a colored formazan using a microplate reader (Synergy HT, Biotek, Winooski, VT, USA). The data were expressed in percentage of control (i.e., optical density of formazan from cells not exposed to PVP formulations).

The dark toxicity of **PVP-2,ab** and **PVP-3a,b** formulations was evaluated under the same protocol, though without the irradiation procedure.

#### 4.6.3. Statistical Analysis 

The results are presented as mean of at least 3 independent assays with 3 replicates per assay. The statistical analysis was performed with GraphPad Prism (GraphPad Software, San Diego, CA, USA). Statistical significance among the conditions was assessed using the nonparametric Mann–Whitney test.

## 5. Conclusions

In summary, two different approaches to prepare mono-charged benzoporphyrin-based Ps were efficiently developed. The reaction of the neutral precursors with (2,2′-bipyridine)dichloroplatinum(II) allows preparing the corresponding benzoporphyrin-platinum(II) modified at the isoindole-type unit in good-to-excellent yields, while the alkylation with iodomethane gives the cationic benzoporphyrins in almost quantitative yields. All the mono-cationic benzoporphyrin derivatives prepared were successfully incorporated in PVP micelles, allowing to improve their solubility in aqueous medium.

Both compounds and PVP-PSs formulations display photophysical features typical of free-base porphyrin derivatives, which are not noticeably affected by the different moieties inserted into the benzoporphyrin core. The PVP-PSs formulations prepared are stable when irradiated with white light and all are able to generate singlet oxygen. However, the PVP formulations prepared with the benzoporphyrin-platinum(II) derivatives exhibit better performance in the ^1^O_2_ generation.

Under the context of PDT evaluation, **PVP-3a,b** formulations demonstrated higher photostability, higher internalization into the cancer cell line, and, consequently, were the most active PSs causing a decrease in HT-1376 cell viability higher than the corresponding formulations with benzoporphyrin-platinum(II) derivatives. Moreover, the synthetic approach to prepare the mono-cationic derivatives **3a,b** exhibits a much better cost-effectiveness relationship when compared with the route to prepare the corresponding derivatives **2a,b**, due to affording almost quantitative yields for both compounds, as well as the high cost associated with (2,2′-bipyridine)chloroplatinum(II) or its synthesis.

Additionally, none of the formulations tested exhibit dark toxicity for HT-1376 cell line, suggesting that the phototoxic effect is due to the reactive oxygen species production under irradiation. These promising results encourage further in vitro and in vivo test studies of benzoporphyrin derivatives as prototypes of future PDT agents.

## Figures and Tables

**Table 1:** Photophysical data of **2a,b** and **3a,b** and PVP-PS formulations **PVP-2a,b** and **PVP-3a,b** in DMF at 298 K.

Compd	λ_max_(nm):log ε	λ_em_ (nm)	Stokes Shift (cm^−1^)	Φ_F_
**2a**	286:4.44 312:4.43 323:4.45 427:5.45 518:4.01 593:3.57	659, 719	151,515.2	0.07
**2b**	312:4.67 324:4.43 427:5.49 520:4.32 594:3.87	660, 720	3,151,515.2	0.06
**3a**	427:5.39 518:4.07 593:3.72	659, 720	151,515.2	0.06
**3b**	429:5.06 522:4.15 597:3.74	662, 723	153,846.2	0.05
**PVP-2a**	282:4.87 312:4.80 324:4.84 426:5.45 518:3.95 595:3.52	658, 719	158,730.2	0.07
**PVP-2b**	285:4.53 309:4.57 323:4.56 426:5.08 519:4.04 594:3.58	661, 720	149,253.7	0.04
**PVP-3a**	427:4.99 520:3.82 595:3.47	658, 720	158,730.2	0.05
**PVP-3b**	428:3.91 523:3.91 597:3.52	662, 725	153,846.2	0.04

**Table 2:** IC50_PDT_ (μM) values of **PVP-2a,b** and **PVP-3a,b** formulations in HT-1376 cell line (for a fluence rate of 20 mW·cm^−2^).

PVP Formulation	IC50_PDT_ *
**PVP-2a**	6.42 μM
**PVP-2b**	8.14 μM
**PVP-3a**	5.58 μM
**PVP-3b**	5.51 μM

* Calculated by extrapolation from the non-linear regression curve MTT reduction (% from control) vs. log(concentration).

## Data Availability

The data presented in this study are available in the Supplementary Materials.

## Supplementary Materials

The following are available online, Figures S1–S22: copies of ^1^H, ^13^C, 2D NMR, and MS spectra of compounds **2a,b** and **3a,b**; Figure S23: UV-Vis, emission, and excitation spectra of compounds **2a** and **3b**, and PVP formulations **PVP-2b** and **PVP-3b**; Figure S24: Dark toxicity of formulations **PVP-2a,b** and **PVP-3a,b** in HT-1376 cells; Table S1: photostability data of PVP formulations.

## Sample Availability

Samples of compounds **1**–**3** are available from the authors.
