# Supplementary material for: Unraveling the Photodynamic Activity of Cationic Benzoporphyrin-Based Photosensitizers against Bladder Cancer Cells
Source: Molecules. 2021 Sep 1;26(17):5312. doi: 10.3390/molecules26175312 (PMC8434352; doi:10.3390/molecules26175312)
Supplement: Supplementary file 1 [file molecules-26-05312-s001.zip › molecules-1344415-supplementary.pdf]

molecules

MDPI

Suporting Information

Unraveling the Photodynamic Activity of Cationic Benzoporphyrin-Based Photosensitizers against Bladder Cancer Cells

Ana T. P. C. Gomes [1,2,3,4,*], M. Graça P. M. S. Neves [1], Rosa Fernandes [2,3], Carlos F. Ribeiro [2,3], José A. S. Cavaleiro [1,*] and Nuno M. M. Moura [1,*]

1 LAQV-REQUIMTE, Department of Chemistry, University of Aveiro, 3810-193 Aveiro, Portugal; gneves@ua.pt
2 Coimbra Institute for Clinical and Biomedical Research (iCBR), Faculty of Medicine, University of Coimbra, 3000-548 Coimbra, Portugal; rcfernandes@fmed.uc.pt (R.F.); cribeiro@fmed.uc.pt (C.F.R.)
3 Center for Innovative Biomedicine and Biotechnology (CIBB), University of Coimbra, 3004-504 Coimbra, Portugal
4 Center for Interdisciplinary Research in Health (CIIS), Faculty of Dental Medicine, Universidade Católica Portuguesa, 3504-505 Viseu, Portugal
* Correspondence: ana.peixoto@ua.pt (A.T.P.C.G.); jcavaleiro@ua.pt (J.A.S.C.); nmoura@ua.pt (N.M.M.M.); Tel.: +351-234- (J.A.S.C.)

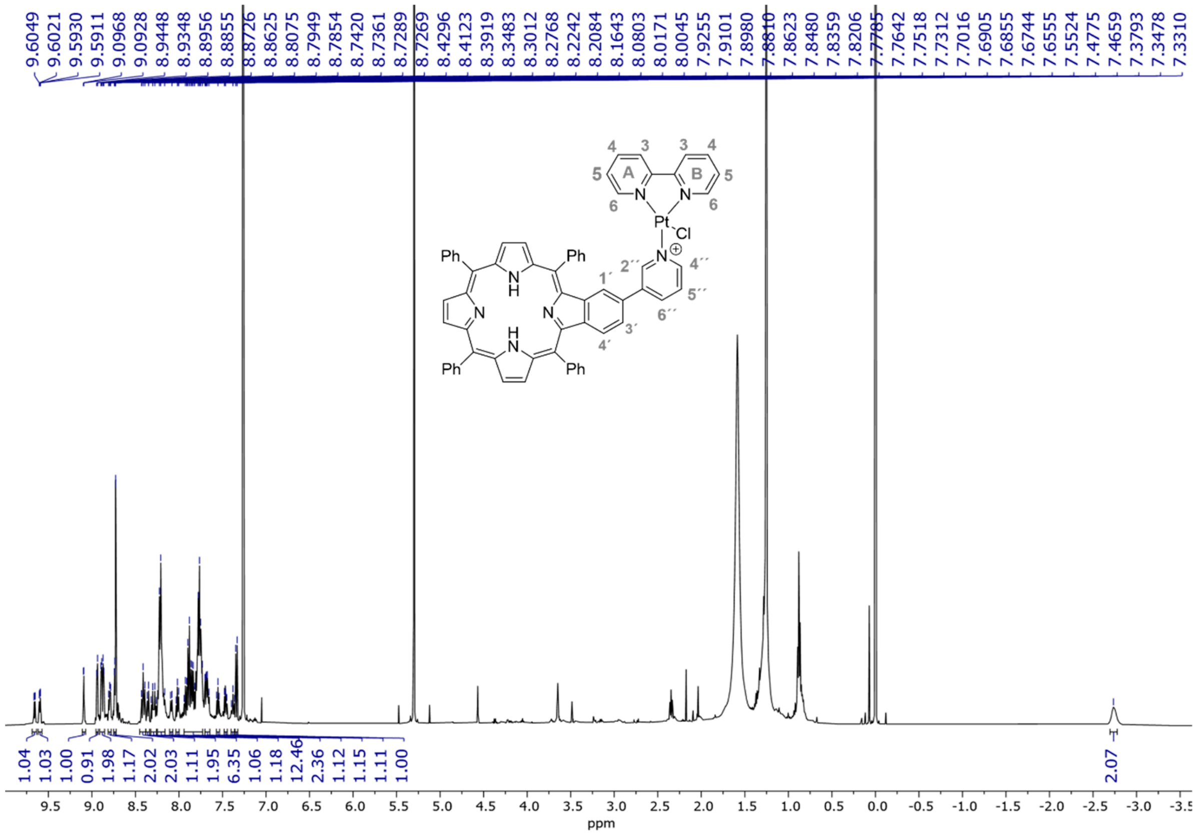

Figure S1. ^{1}H NMR spectrum of compound **2a** in $CDCl_3$.

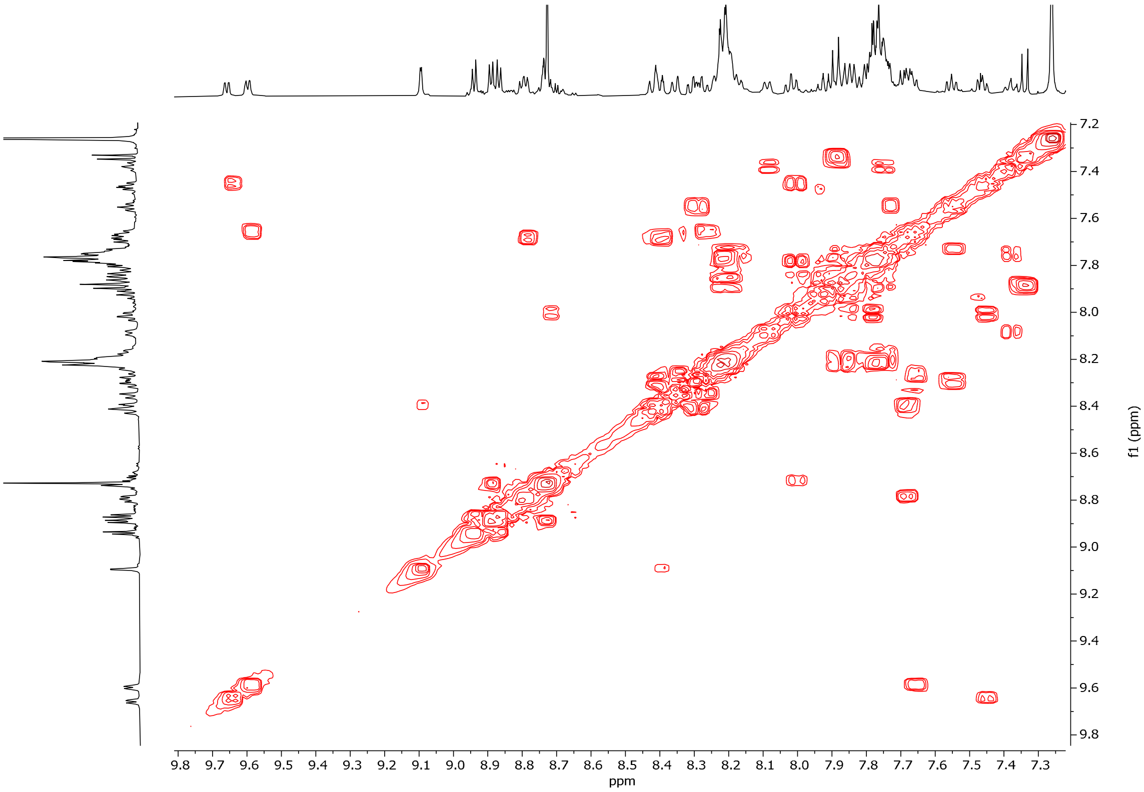

Figure S2. Partial $^{1}H/^{1}H$ COSY spectrum of compound **2a** in $CDCl_3$.

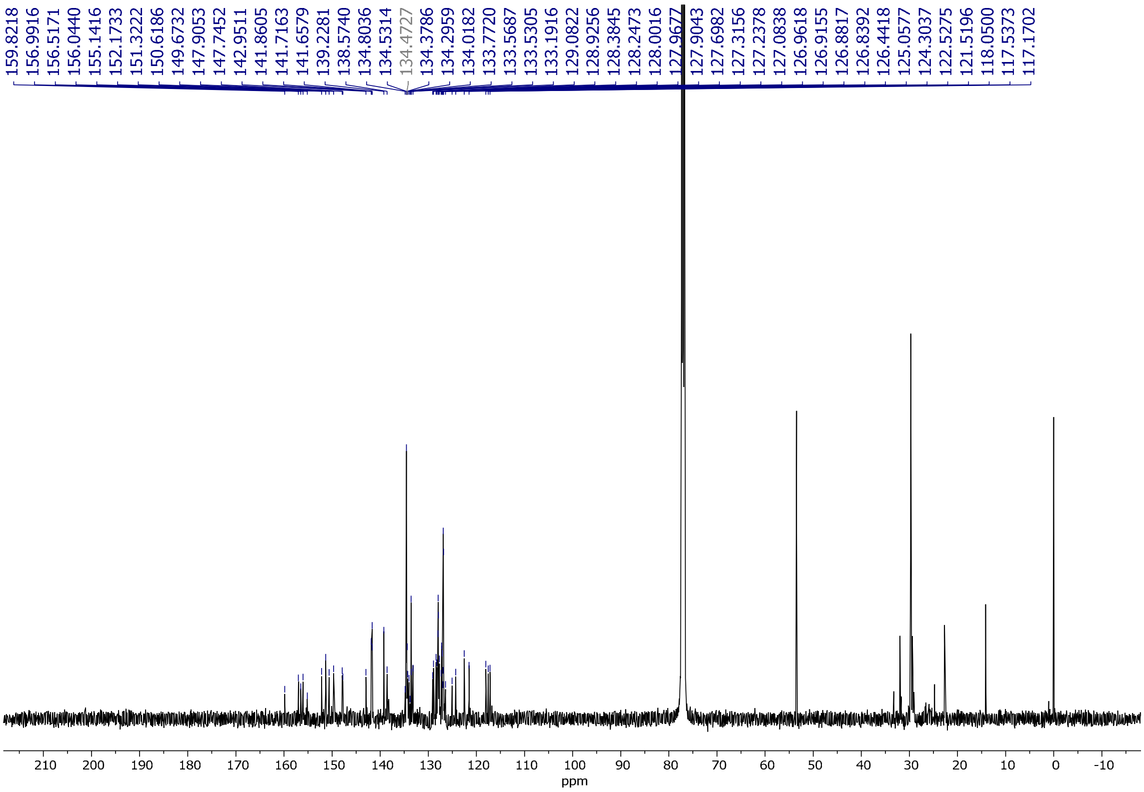

Figure S3. ^{13}C NMR spectrum of compound **2a** in $CDCl_3$.

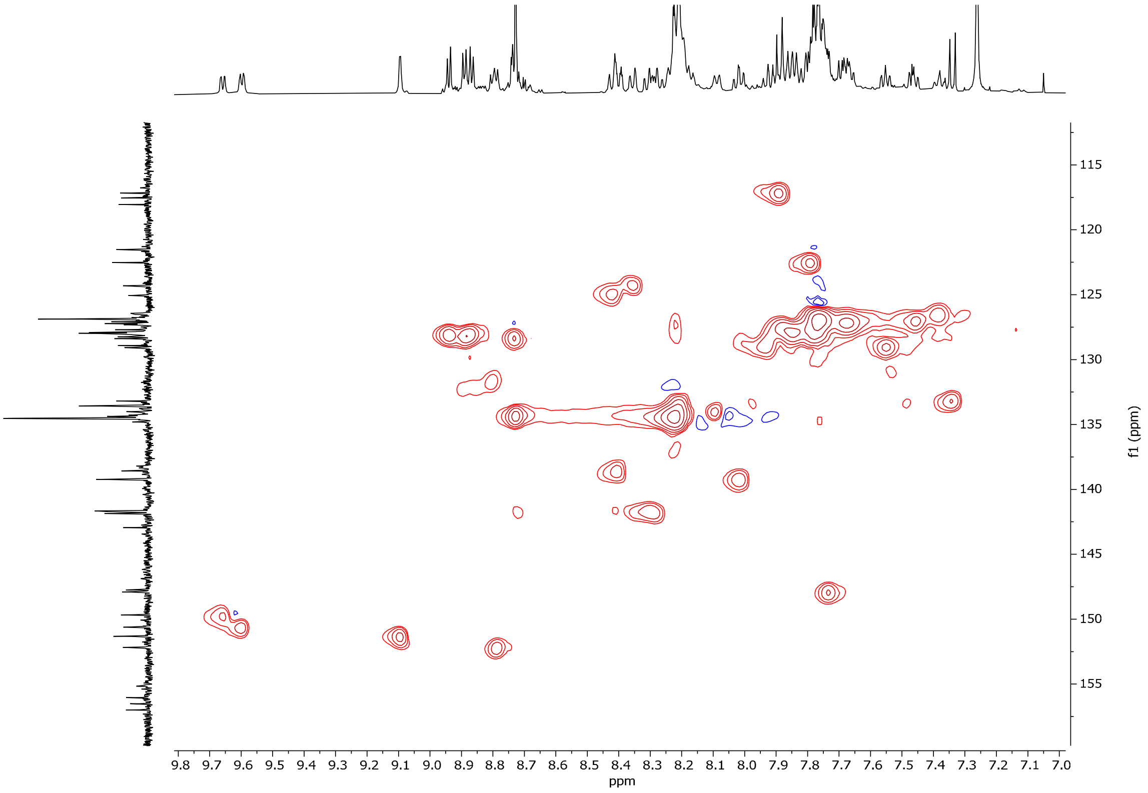

Figure S4. Partial $^{1}H/^{13}C$ HSQC spectrum of compound **2a** in $CDCl_3$.

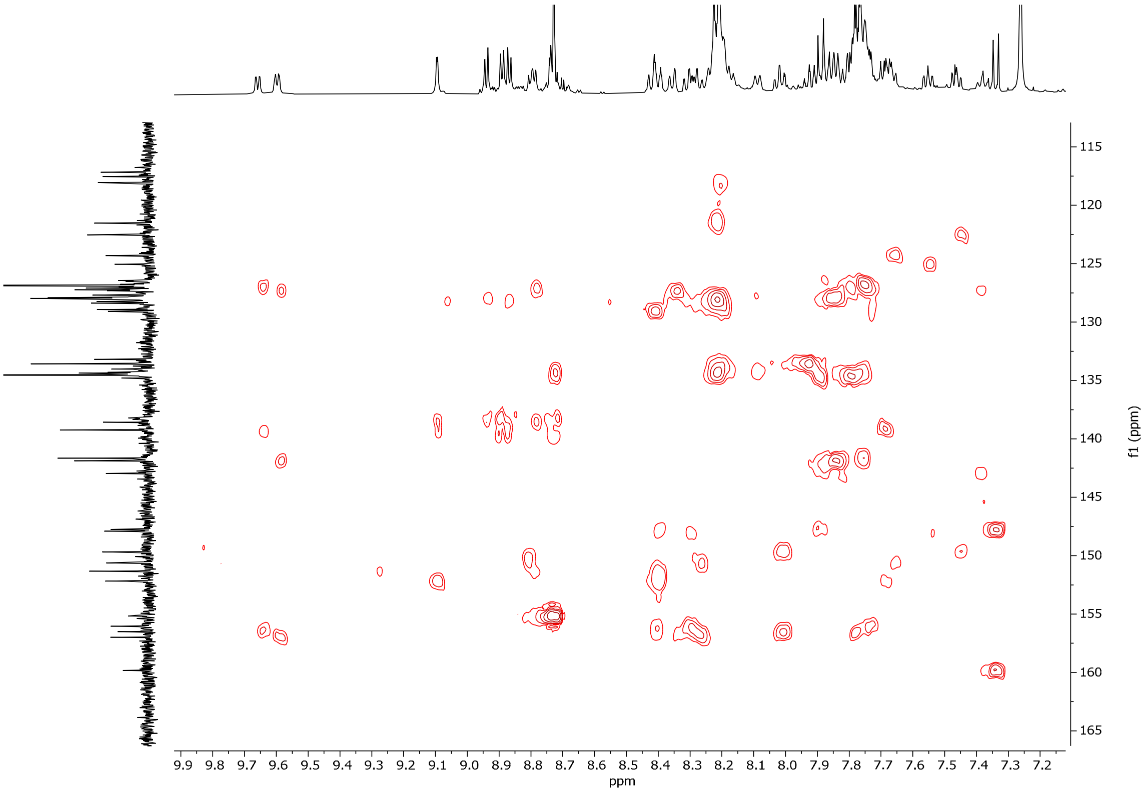

Figure S5. Partial $^{1}H/^{13}C$ HMBC spectrum of compound **2a** in $CDCl_3$.

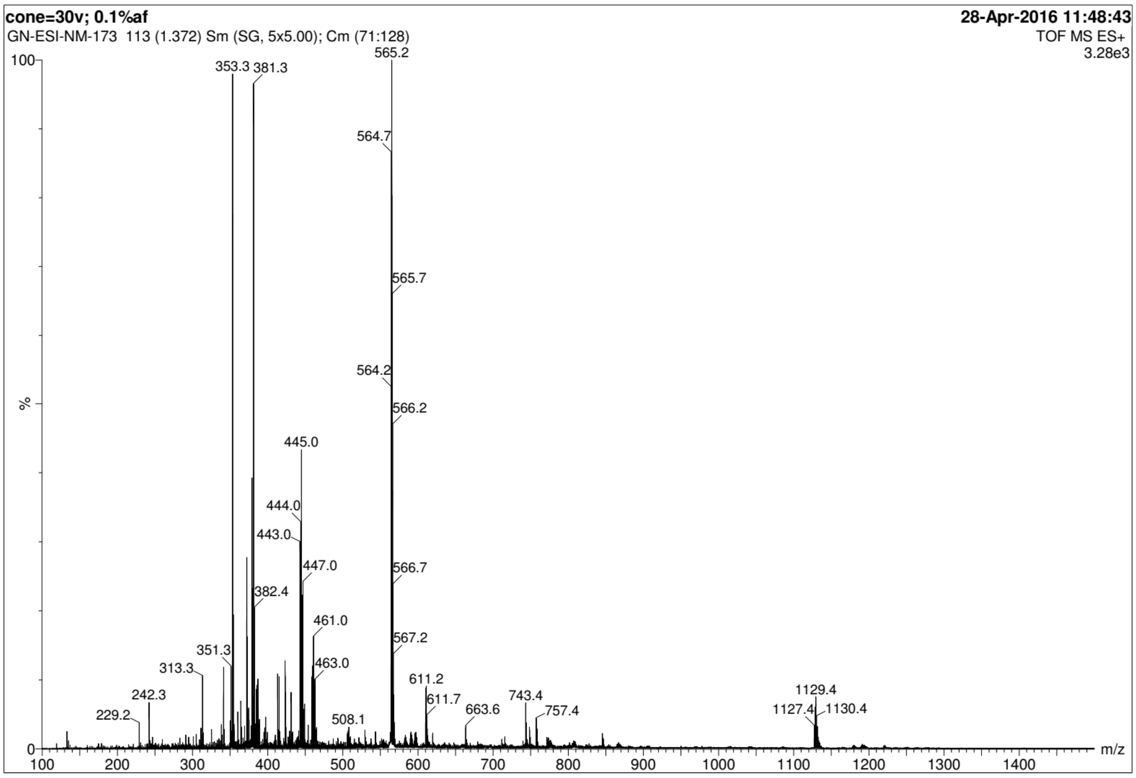

Figure S6. MS-ESI(+) spectrum of compound **2a**.

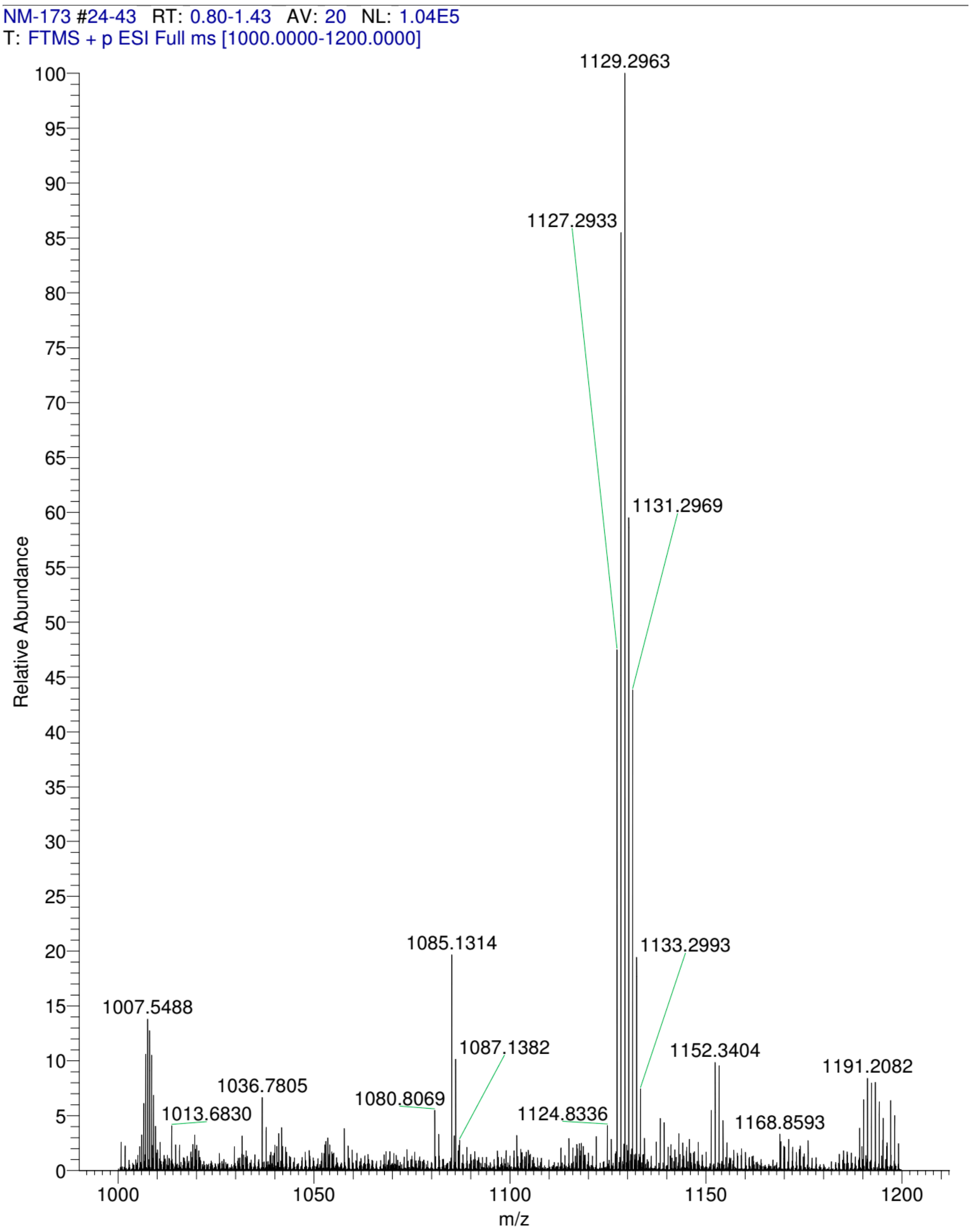

Figure S7. HRMS-ESI(+) spectrum of compound **2a**.

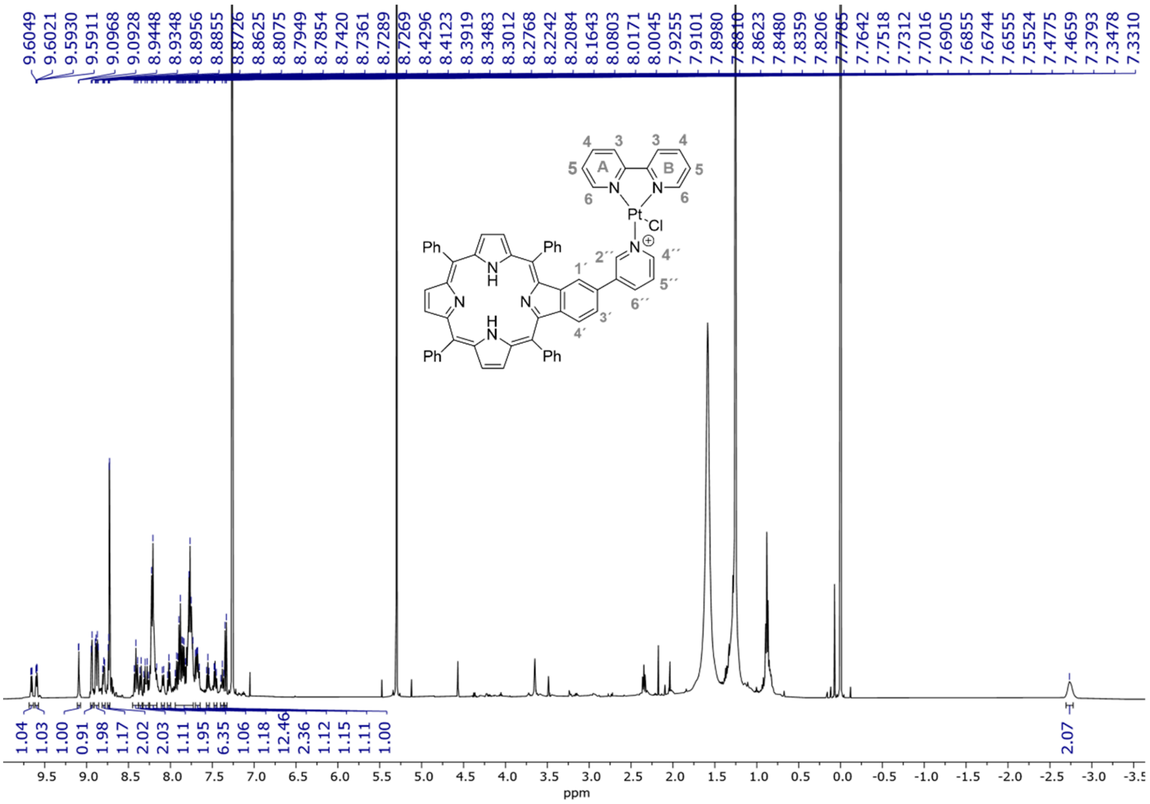

Figure S8. ^{1}H NMR spectrum of compound **2b** in $CDCl_3$.

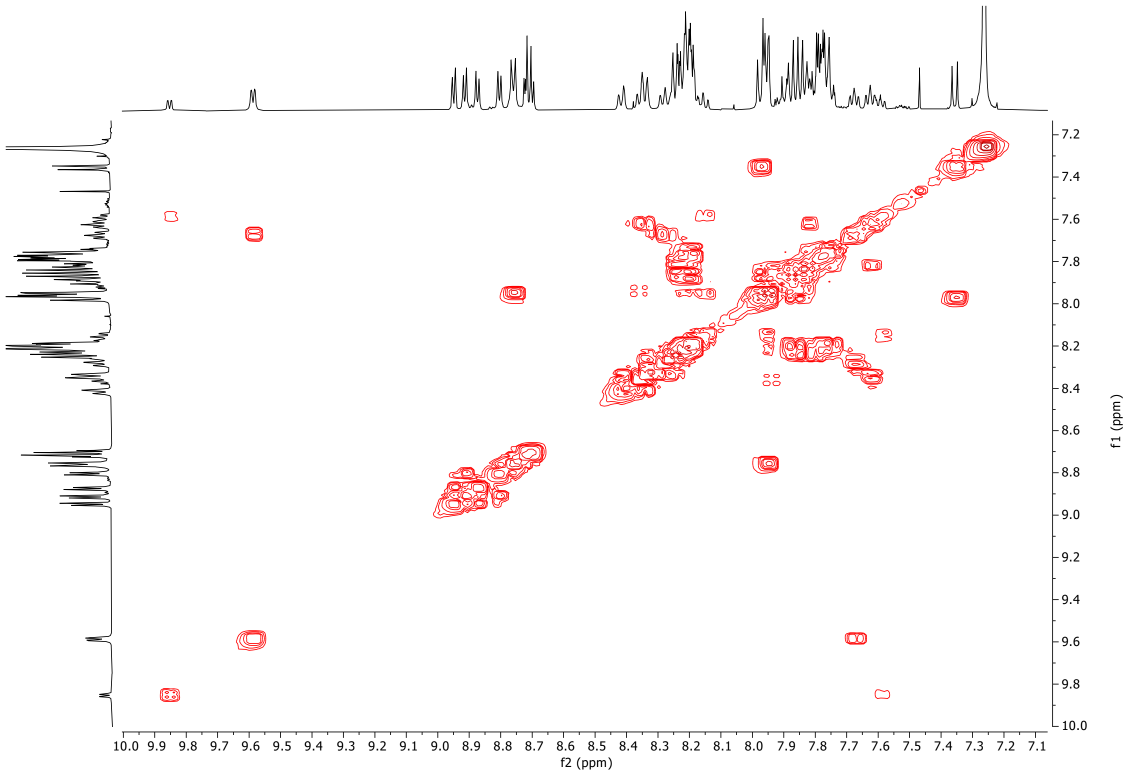

Figure S9. Partial ^{1}H/^{1}H COSY spectrum of compound **2b** in $CDCl_3$.

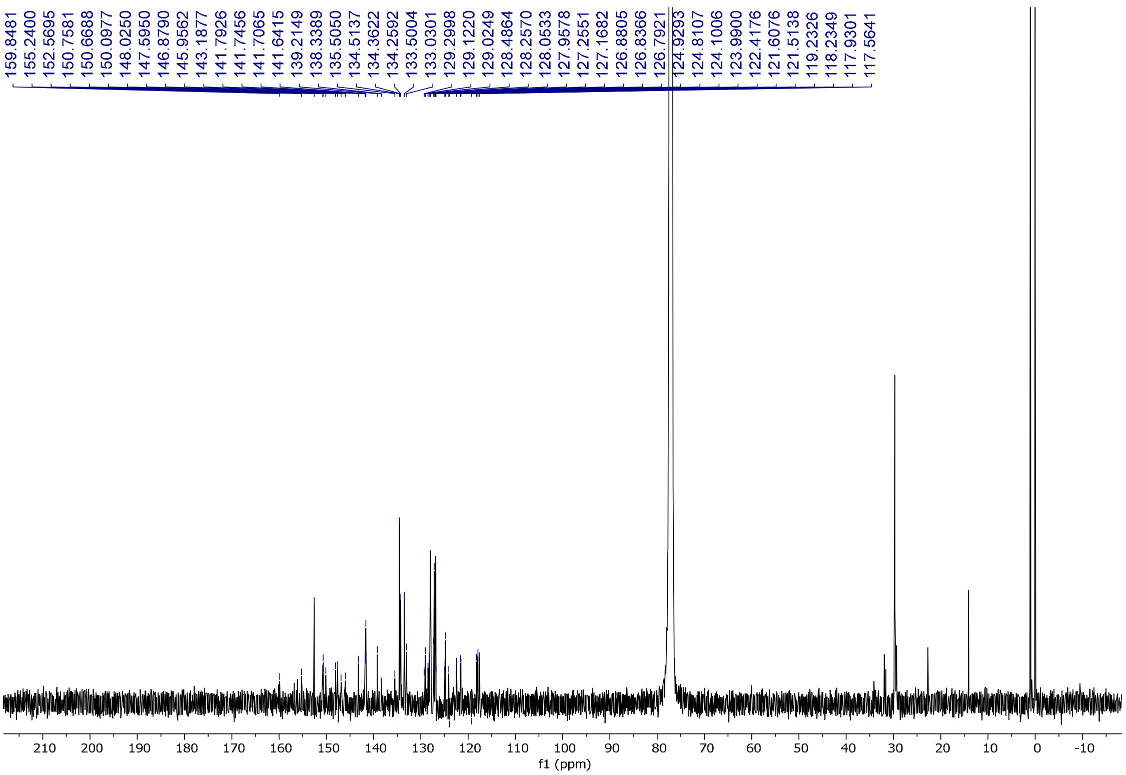

Figure S10. ^{13}C NMR spectrum of compound **2b** in $CDCl_3$.

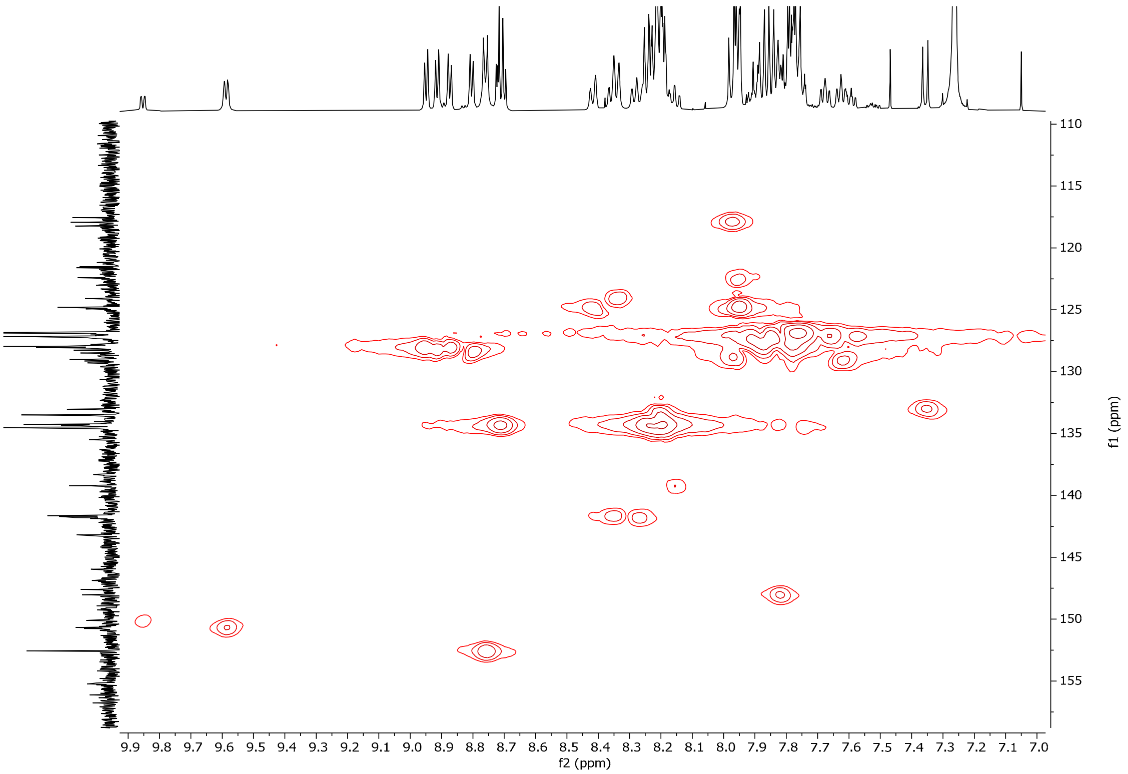

Figure S11. Partial $^{1}H/^{13}C$ HSQC spectrum of compound **2b** in $CDCl_3$.

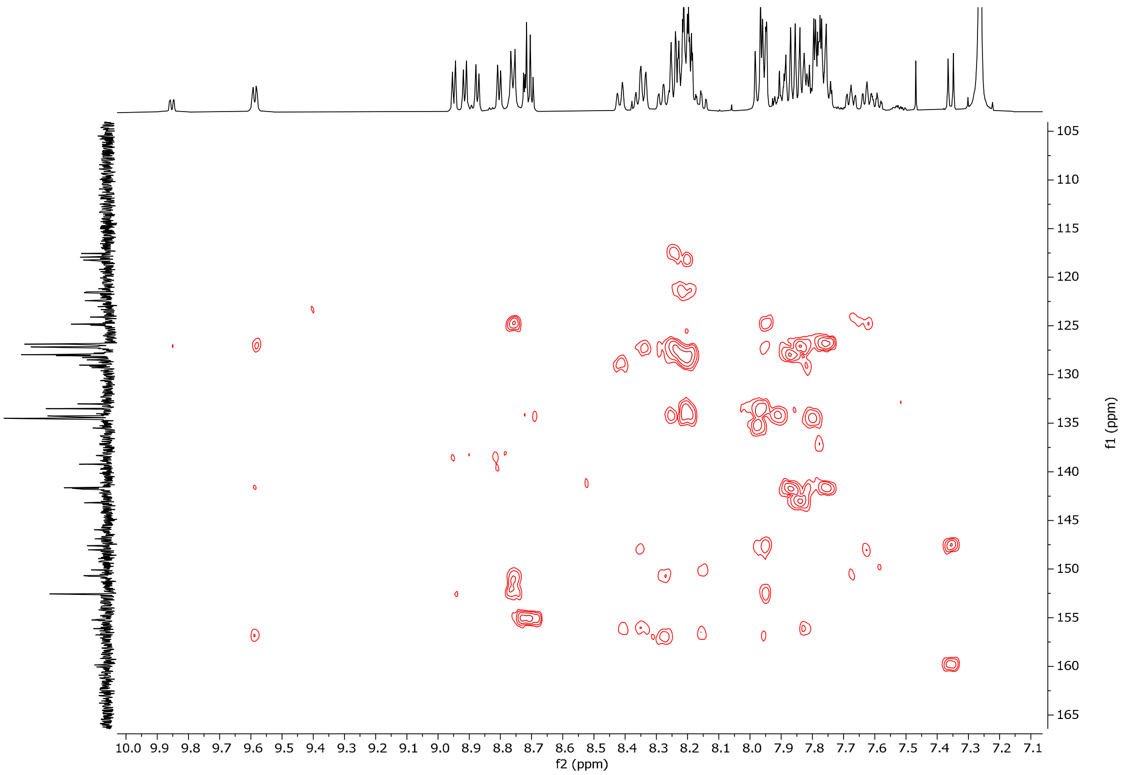

Figure S12. Partial $^{1}H/^{13}C$ HMBC spectrum of compound **2b** in $CDCl_3$.

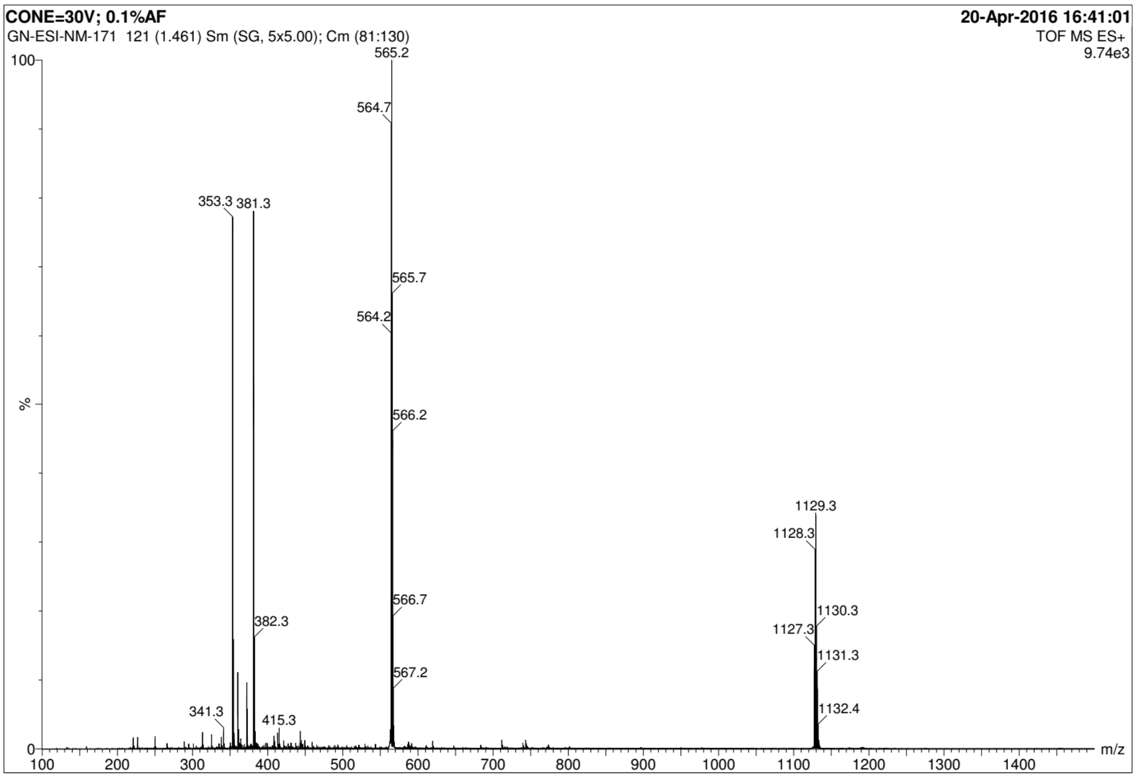

Figure S13. MS-ESI(+) spectrum of compound **2b**.

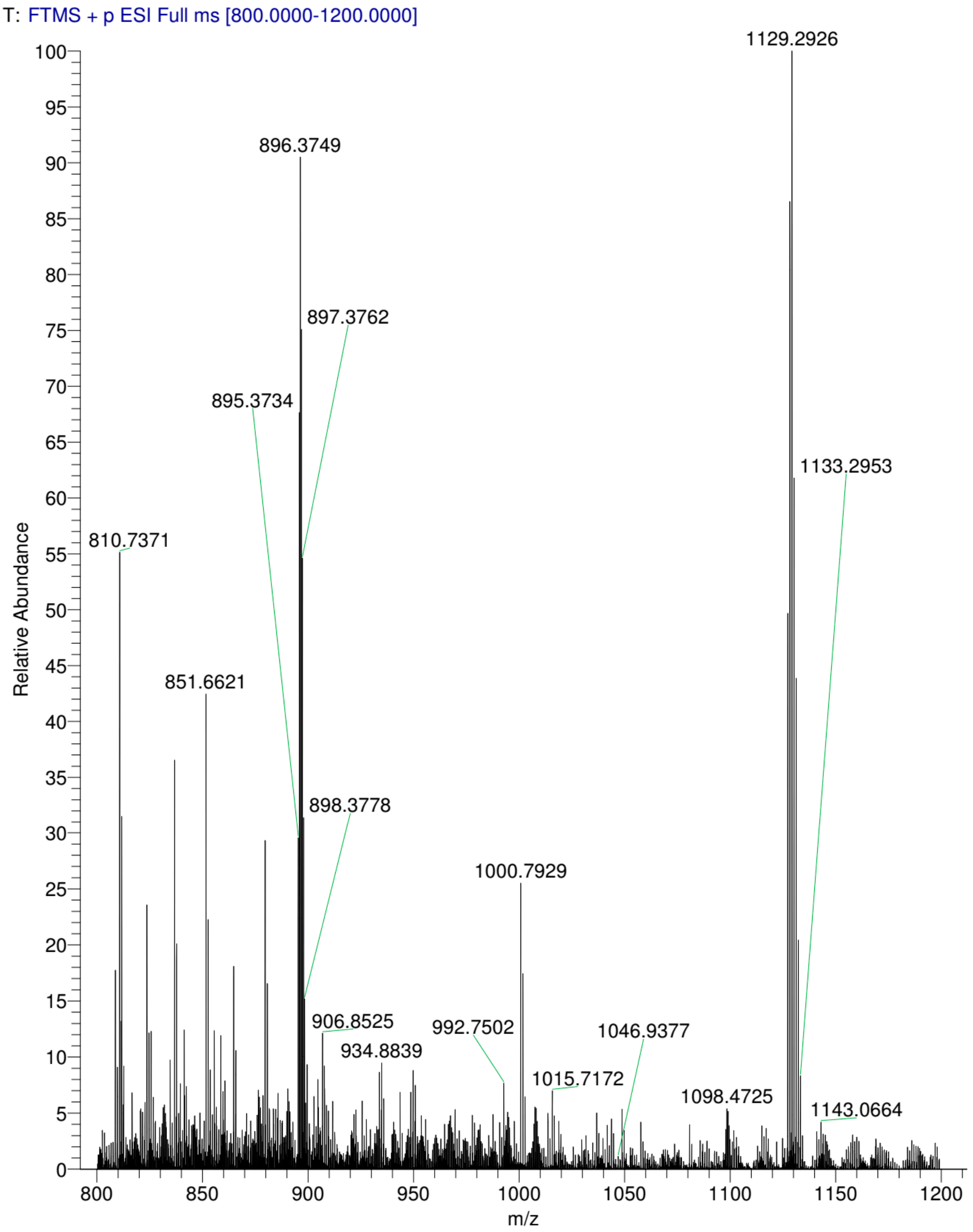

Figure S14. HRMS-ESI(+) spectrum of compound **2b**.

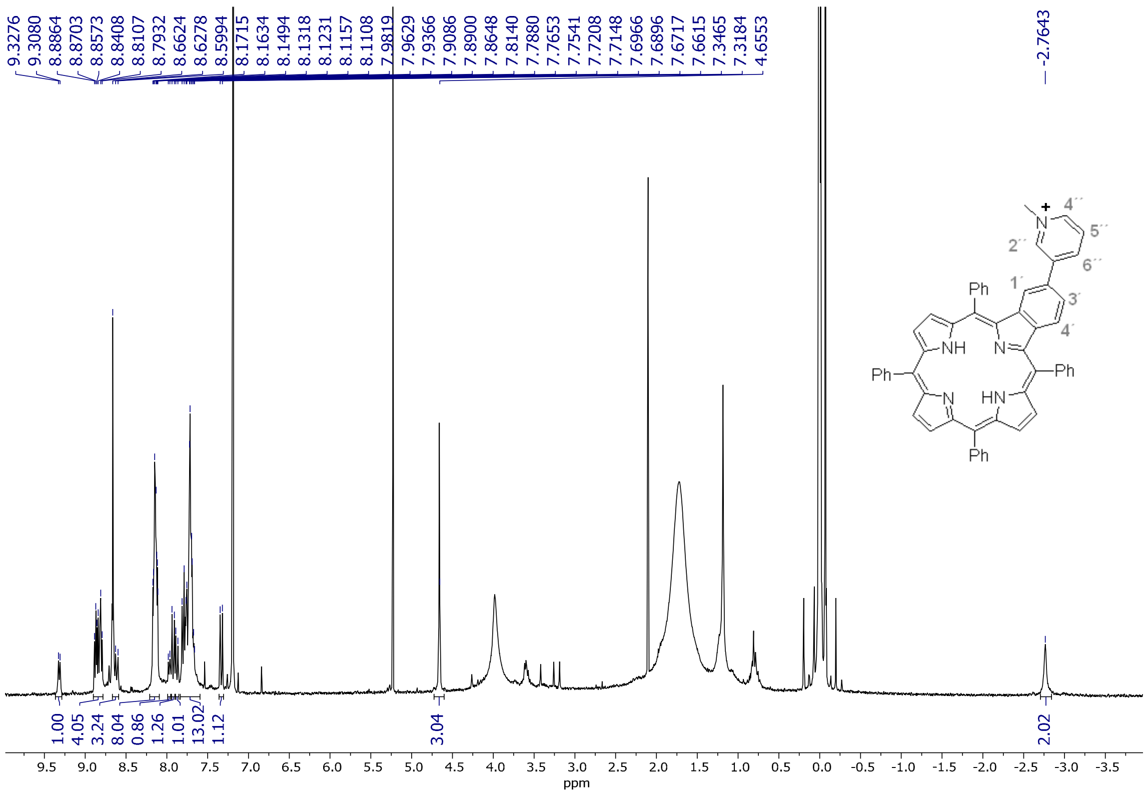

Figure S15. ^{1}H NMR spectrum of compound **3a** in $CDCl_3$.

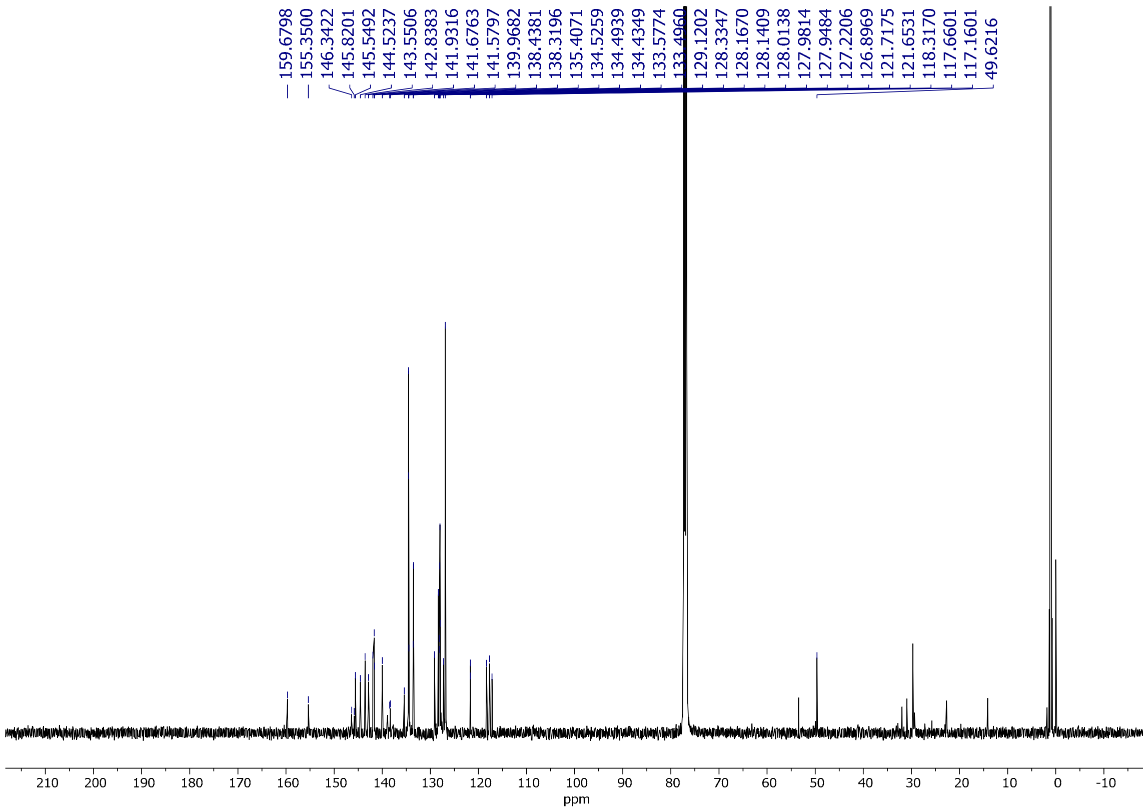

Figure S16. ^{13}C NMR spectrum of compound **3a** in $CDCl_3$.

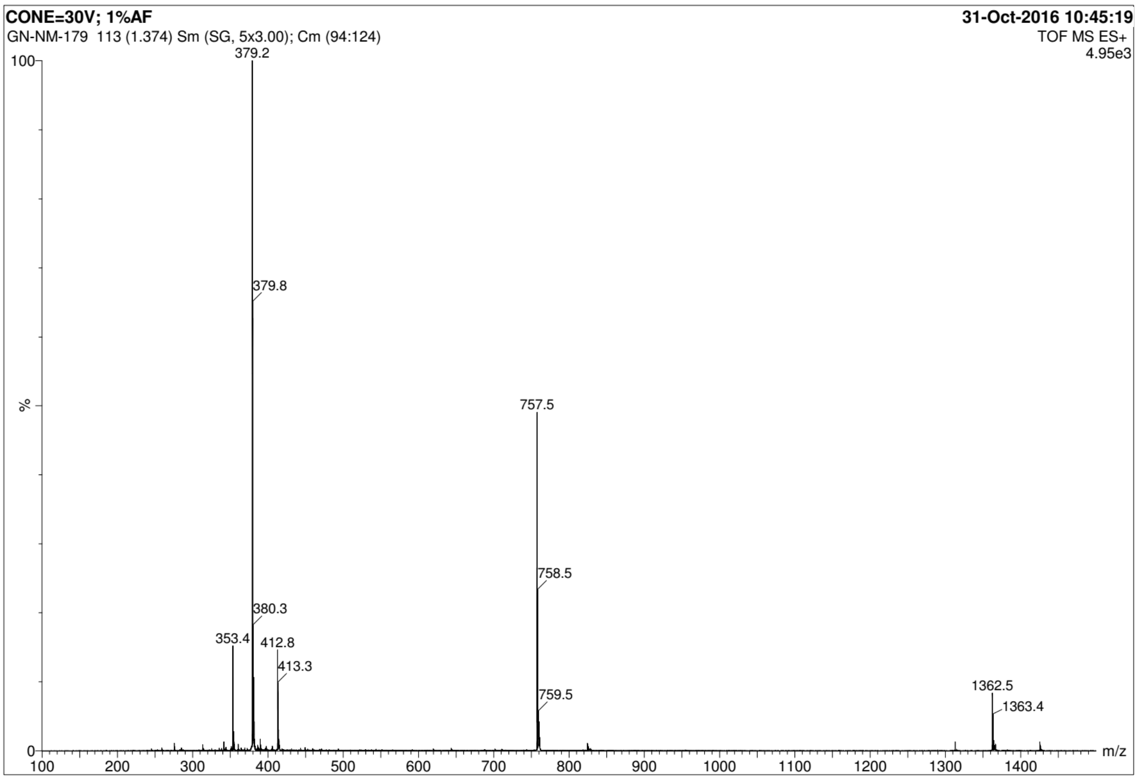

Figure S17. MS-ESI(+) spectrum of compound **3a**.

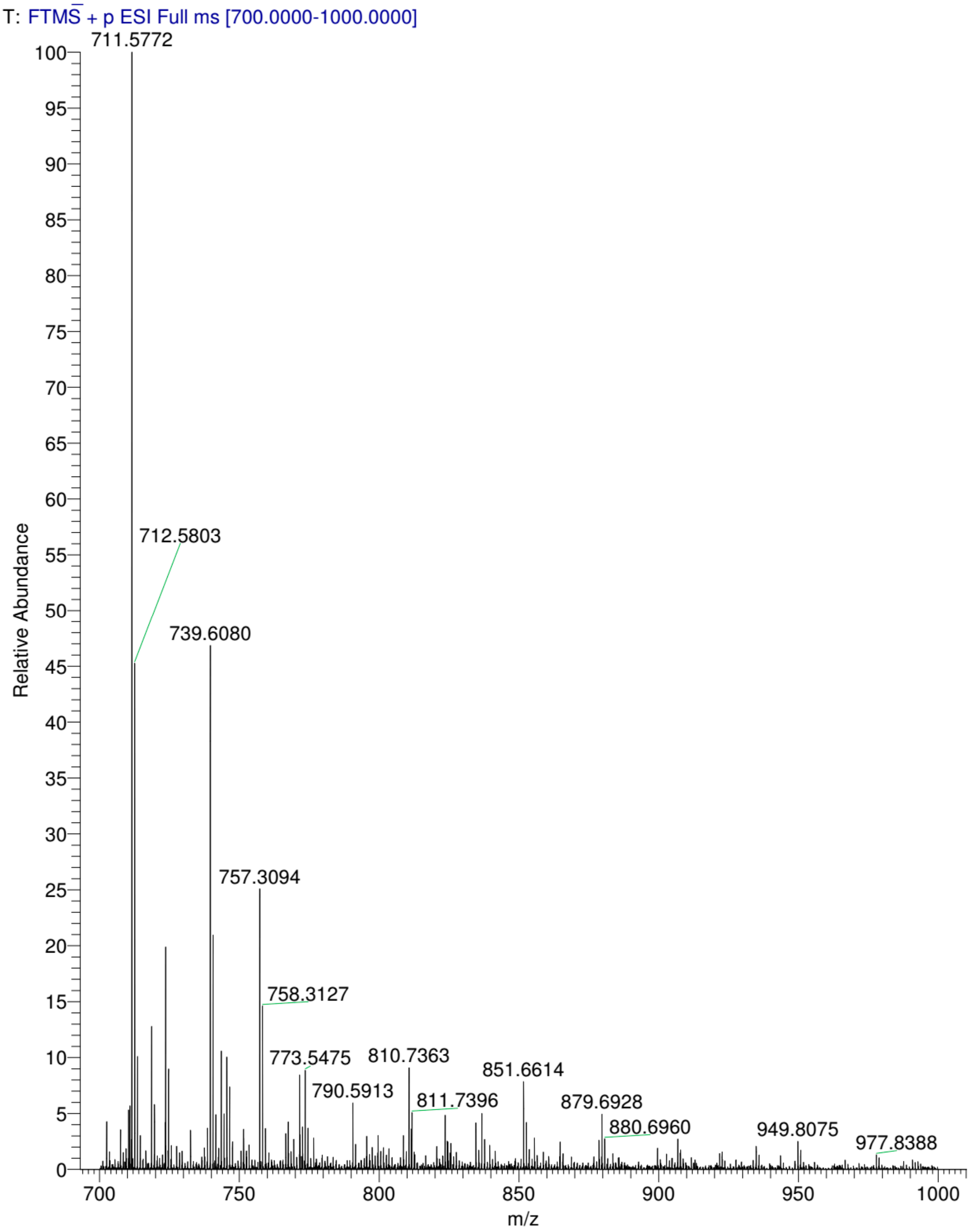

Figure S18. HRMS-ESI(+) spectrum of compound **3a**.

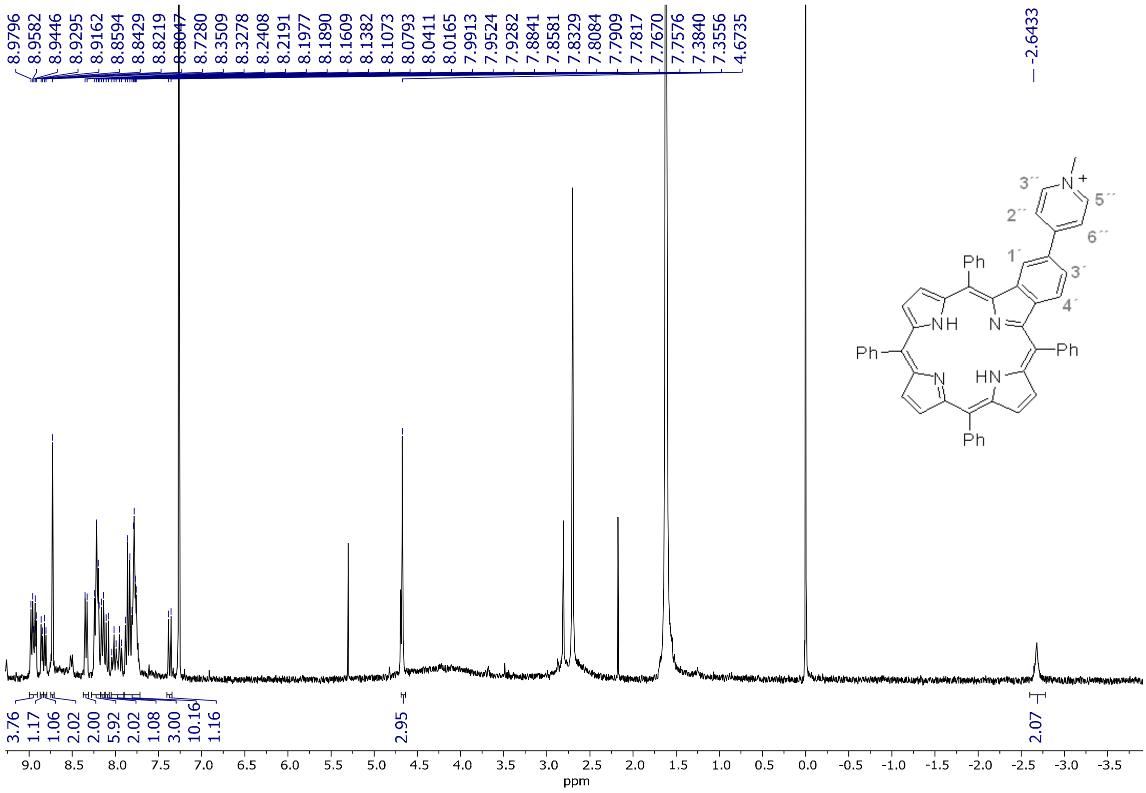

Figure S19. ^{1}H NMR spectrum of compound **3b** in $CDCl_3$.

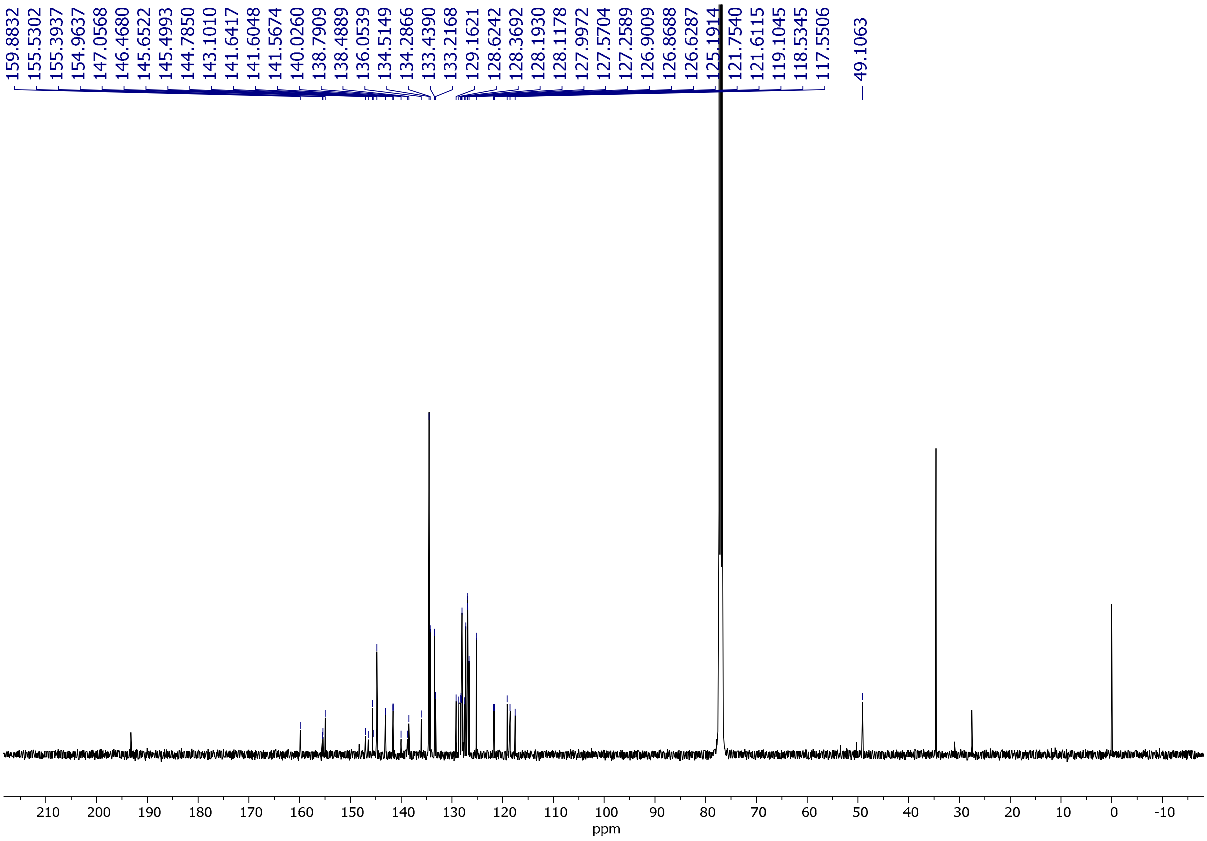

Figure S20. ^{13}C NMR spectrum of compound **3b** in $CDCl_3$.

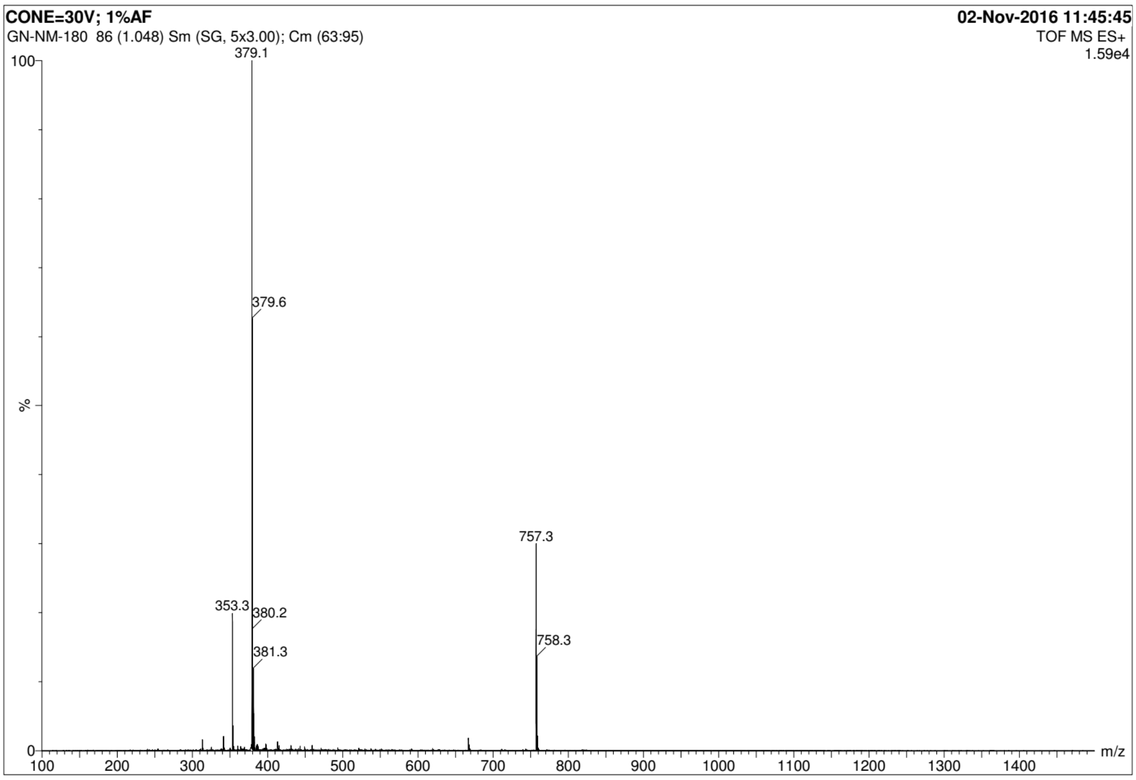

Figure S21. MS-ESI(+) spectrum of compound **3b**.

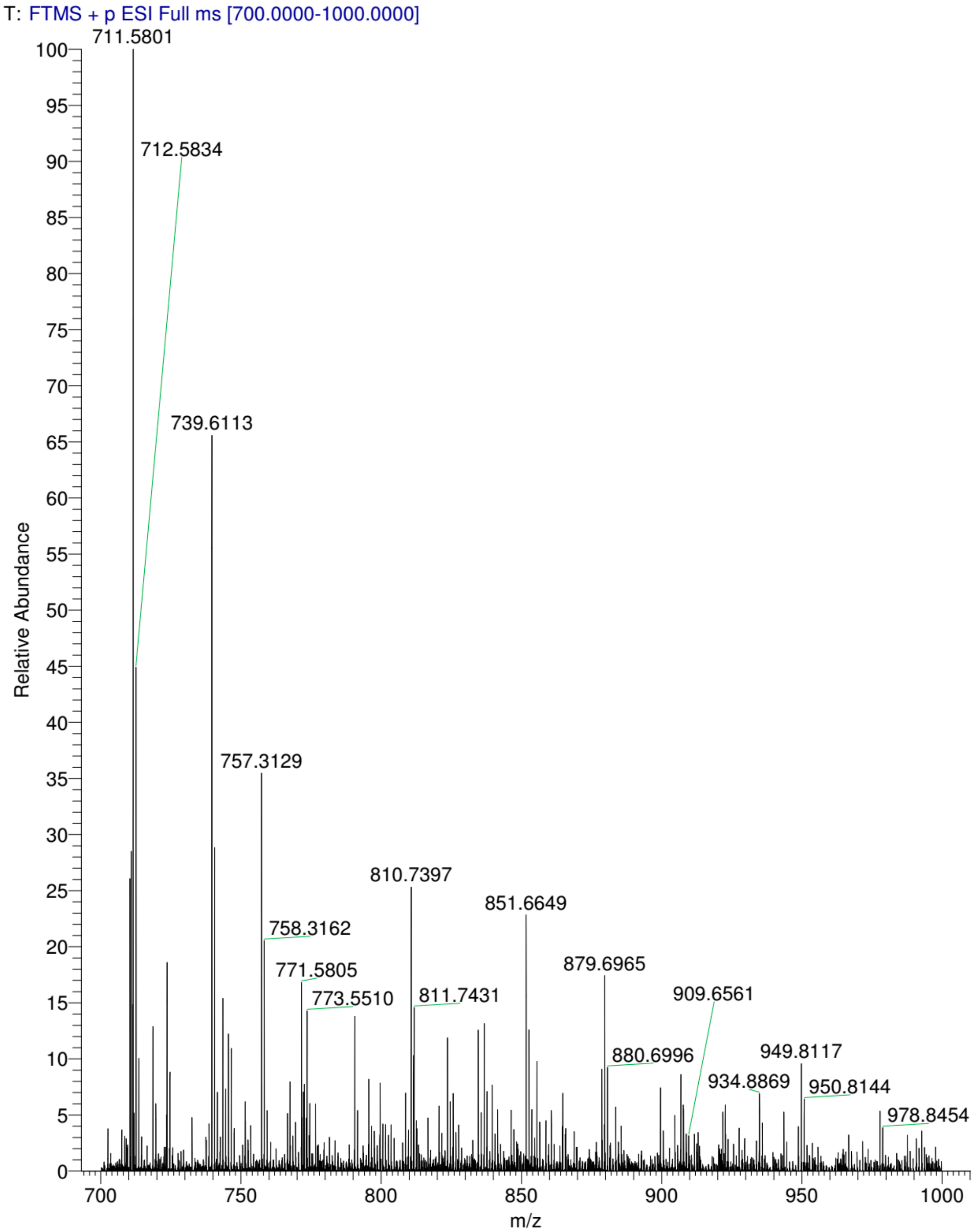

Figure S22. HRMS-ESI(+) spectrum of compound **3b**.

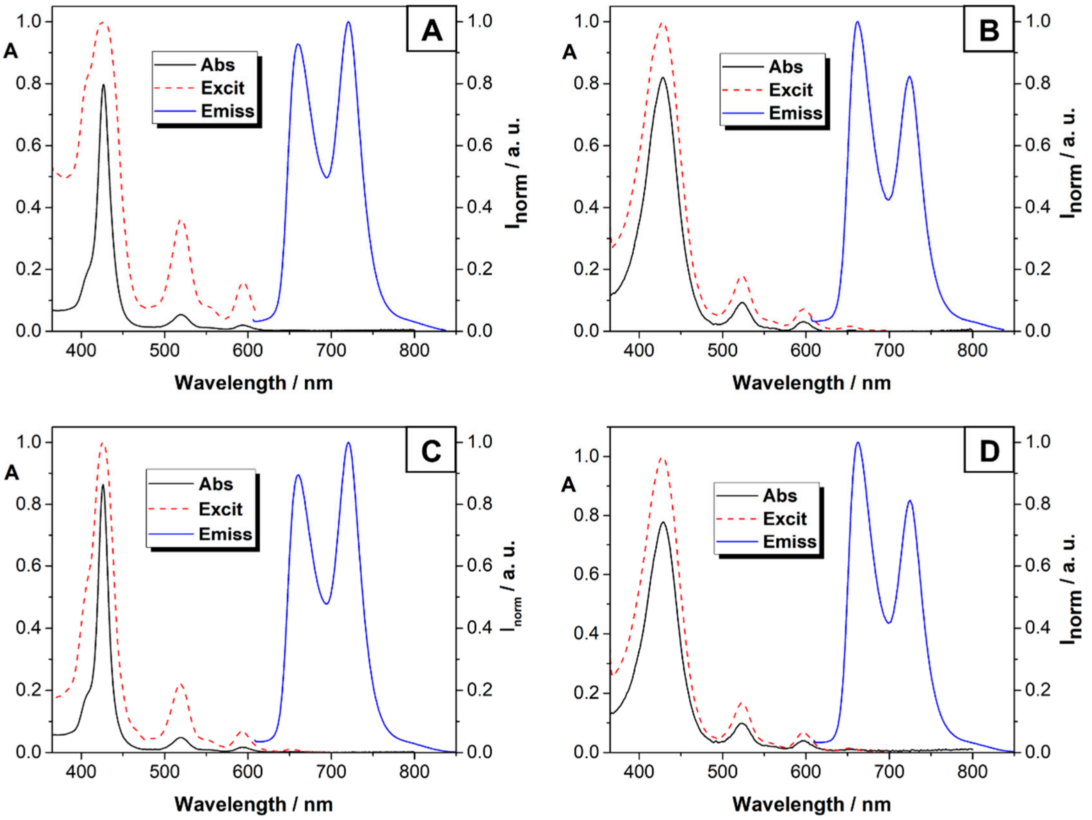

Figure S23. Absorption and normalized emission and excitation spectra of **2b** (A), **3b** (B), **PVP-2b** (B) and **PVP-3b** (B) in DMF at 298 K. ([**2b**] = [**3b**] = [**PVP-2b**] = [**PVP-3b**] = 3.0 x10^{-6} M; $\lambda_{exc\mathbf{2b}}$ = $\lambda_{exc\mathbf{PVP\text{-}2b}}$ = 594 nm and $\lambda_{exc\mathbf{3b}}$ = $\lambda_{exc\mathbf{PVP\text{-}3b}}$ = 597; $\lambda_{em\mathbf{2b}}$ = 660 nm, $\lambda_{em\mathbf{PVP\text{-}2b}}$ = 661 nm and $\lambda_{em\mathbf{3b}}$ = $\lambda_{em\mathbf{PVP\text{-}3b}}$ = 662).

Table S1. Photostability of 5 µM of PVP-PS formulations, after irradiation with white light at a fluence rate of 25 mW.cm^{-2} for different periods of time (0-30 min).*

PS	λ_{max} (nm)	Irradiation time (min)								
		**0**	**1**	**2**	**3**	**4**	**5**	**10**	**20**	**30**
**2a+PVP**	426	0	1	2	3	5	7	10	14	16
**2b+PVP**	426	0	1	3	6	9	11	18	23	28
**3a+PVP**	427	0	2	3	3	5	7	9	11	12
**3b+PVP**	428	0	0	0	2	3	3	7	7	11

* The results are presented in percentage calculated by the ratio of residual absorbance at λ_{max} at different periods of time and absorbance before irradiation.

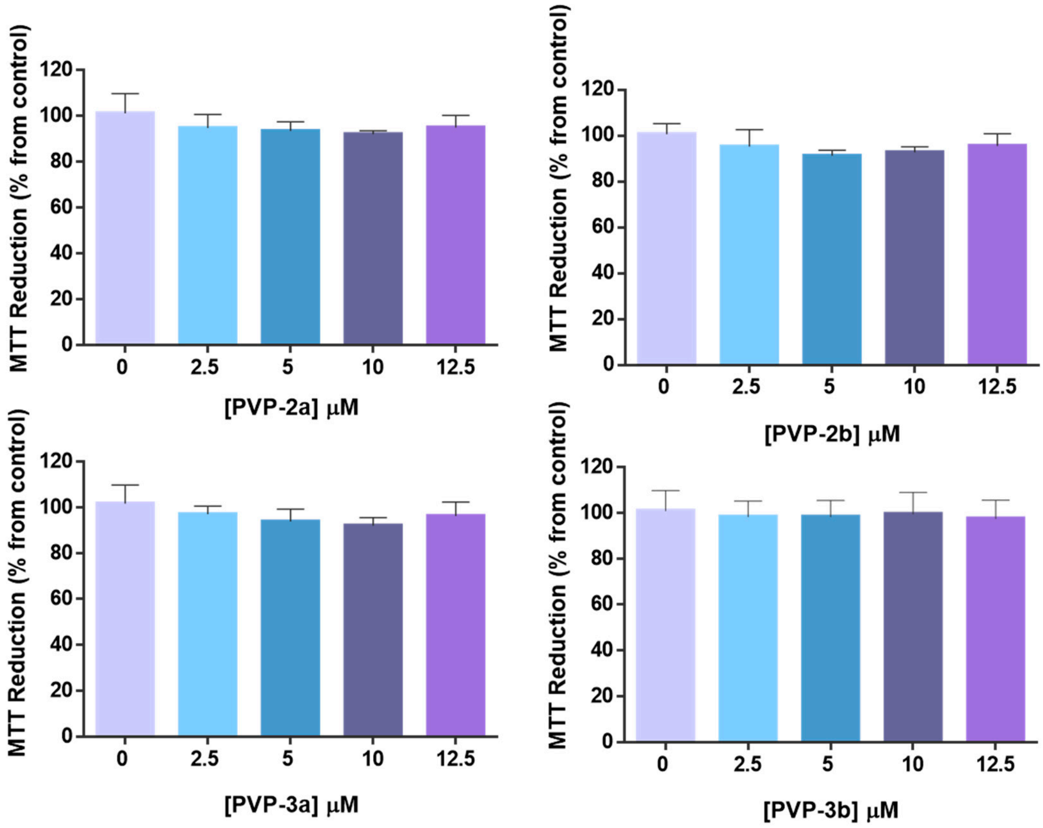

Figure S24. Dark toxicity of formulations **PVP-2a,b** and **PVP-3a,b** in HT-1376 cells. Cells were incubated with the formulations for 4 h in the dark. Cytotoxicity was evaluated 24 h after PDT. Data are the mean value ± S.D. of at least three independent experiments performed in triplicates: *($p < 0.05$), **($p < 0.01$), ***($p < 0.001$) compared to MTT reduction (%) at 24 h after PDT for control cells (untreated cells).
